# Multi-omic analysis reveals dynamic changes of three-dimensional chromatin architecture during T cell differentiation

**DOI:** 10.1038/s42003-023-05141-1

**Published:** 2023-07-24

**Authors:** Ge Zhang, Ying Li, Gang Wei

**Affiliations:** CAS Key Laboratory of Computational Biology, Shanghai Institute of Nutrition and Health, University of Chinese Academy of Sciences, Chinese Academy of Sciences, Shanghai, 200031 China

**Keywords:** Immunology, Data integration, Epigenetics

## Abstract

Cell differentiation results in widespread changes in transcriptional programs as well as multi-level remodeling of three-dimensional genome architecture. Nonetheless, few synthetically investigate the chromatin higher-order landscapes in different T helper (Th) cells. Using RNA-Seq, ATAC-Seq and Hi-C assays, we characterize dynamic changes in chromatin organization at different levels during Naive CD4^+^ T cells differentiation into T helper 17 (Th17) and T helper 1 (Th1) cells. Upon differentiation, we observe decreased short-range and increased extra-long-range chromatin interactions. Although there is no apparent global switch in the A/B compartments, Th cells display the weaker compartmentalization. A portion of topologically associated domains are rearranged. Furthermore, we identify cell-type specific enhancer-promoter loops, many of which are associated with functional genes in Th cells, such as *Rorc* facilitating Th17 differentiation and *Hif1a* responding to intracellular oxygen levels in Th1. Taken together, these results uncover the general patterns of chromatin reorganization and epigenetic landscapes of gene regulation during T helper cell differentiation.

## Abstract (web summary)

Multi-omic analysis of differentiating naive T cells highlights dynamic changes in three-dimensional chromatin architecture that occur during T helper cell differentiation.

## Introduction

In response to an infection, a wide range of cells in innate and adaptive immune systems are activated, and then they collaborate to control and eliminate invading pathogens^1,2^. In immune response, naive CD4^+^ T cells, whose development is precisely regulated by environment and transcription factors, play a critical role. These cells, in particular, can differentiate into specialized subsets of T helper (Th) cells including Th1, Th2, Th17, follicular helper T (Tfh) and regulatory T (Treg) cells^3,4^. Th17 cells, which produce IL-17 in general, are important in tissue inflammation and autoimmunity like psoriasis, Crohn’s disease and multiple sclerosis^5–8^. RORγt (retinoic-acid-receptor-related orphan receptor gamma t), which is encoded by *Rorc*, a lineage-specific transcription factor, regulates Th17 development and maintains state of Th17 cell differentiation^9,10^. To engage in cellular immunity against intracellular microorganisms, Th1 cells produce IFN-γ^4^. Th1 differentiation is primarily induced by the cytokines IL-12 and IFN-γ and is mediated by the signal transducer and activator of transcription 4 (Stat4), Stat1, and the T box transcription factor T-bet^11–13^. However, Th17 cells can readily switch to other T helper cell programs under certain cytokine conditions, which means the plasticity of Th17 cells makes them convert into other T cells like Th1 and Th2^4^. The immune system has evolved to respond to environmental stimuli.

T cell activation causes context-specific gene expression programs that promote biosynthesis and energy generation, cell cycle progression, and ultimately cell differentiation. Previous research has shown that epigenetic mechanisms play roles in T cell differentiation in the context of the immune microenvironment^14,15^. T cell activation is associated with significant alterations in chromatin structure, as evidenced by a marked remodeling of local or global chromatin structure^16–20^. T cell activation also causes genome-wide changes in genome topology and gene-regulatory interactions in humans^21^. For instance, SATB1-dependent regulatory chromatin loops shape the enhancer network of developing T cells^22^. CTCF binding sites even stabilize mouse Th2 specific long-range enhancer-promoter (E-P) loops^23^. CTCF-mediated insulation enables direct interactions between the MYC promoter and a distal super-enhancer according to studying 3D chromatin landscapes in human T cell acute lymphoblastic leukemia^24^. Though numerous efforts to dissect the epigenomic regulation in various T cells, we still lack integrative knowledge of how alterations in the local and global chromatin structure coordinate gene expression in different T helper cells. Also, there has less knowledge to uncover the conservation of chromatin organization during T cell activation for combining human and mouse resources.

Here, we utilize gene expression quantified by RNA-Seq, local chromatin accessibility measured by ATAC-Seq, and higher-order chromatin architecture captured by Hi-C to dissect the multi-omics changes of T cell differentiation. We compare and integrate the higher-order chromatin structure information of human and mouse T cells. We identify numerous cell-type specific E-P loops to define the epigenetic landscapes upon T cell differentiation, including loci such as *Rorc* in Th17 and *Hif1a* in Th1. Interestingly, a series of analyses shows a trend in which many Th17 characteristics fall between Naive and Th1, which is consistent with the degree of differentiation across three T cells. Therefore, our findings provide a foundational resource and reference to illustrate the chromatin architecture traits during T cell differentiation in-depth.

## Results

### High-quality data from sequenced libraries

T cell differentiation causes extensive epigenetic changes^25–28^. To investigate the chromatin conformation dynamics of Naive differentiation into Th17 and Th1, we first isolated non-activated Naive CD4^+^ T cells (Naive) from mice and differentiated the cells into Th17 and Th1 under the specific polarization conditions. Then, we integrated RNA-Seq, ATAC-Seq and Hi-C assays to characterize the epigenome landscapes during T cell differentiation (Supplementary Fig. 1). Two independent biological replicates were used for each cell line across above three genomics techniques. The results showed that the data were of high quality and reproducibility, including principal component analysis or Pearson correlation analysis of normalized reads at the corresponding omics layers, and reproducibility analysis of the Hi-C data at the compartment and topologically associated domain (TAD) levels (Supplementary Fig. 2 and Supplementary Fig. 3a-b). In brief, a total of ~100 million reads per pooled cell line were mapped for RNA-Seq by Hisat2^29^ with alignment rates ~93% per biology replicate (Supplementary Table 1); 26262, 32662, 35221 chromatin accessible regions were identified by ATAC-Seq in Naive, Th17 and Th1 cells, respectively; and a total of ~101 to ~186 million valid pairs per replicate were generated from the Hi-C data (Supplementary Table 2).

### Widespread changes induced by T cell differentiation in gene expression and chromatin accessibility

**Fig. 1:**
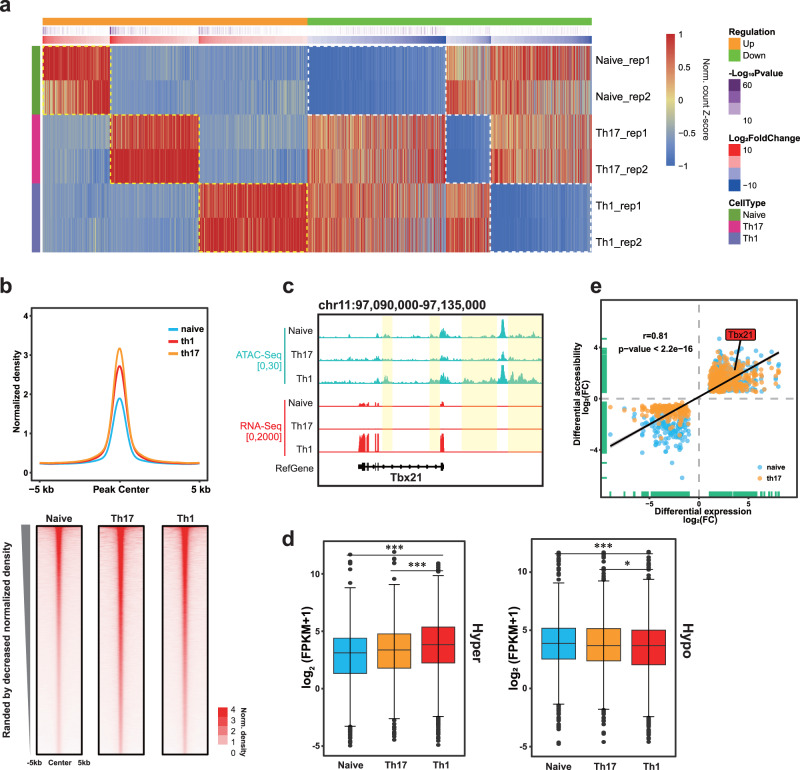
Extensive changes in transcriptome and local chromatin structure caused by T cell differentiation. **a** Heatmap of cell-type specific differentially expressed genes (DEGs) ordered by decreased log_2_FoldChange per group identified from DESeq2. Colors indicate the normalized count z-score in each cell-type specific DEG across all T cell types. DEGs are filtered by adjusted p-value < 0.01 and |log_2_(FoldChange)|>1. Yellow dashed boxes represent cell-type specific up-regulated genes, while white dashed boxes represent cell-type specific down-regulated genes. **b** Profiles of ATAC-Seq normalized density around peak center ±5 kb. **c** The genomic tracks show ATAC-Seq (green) and RNA-Seq (red) signals around *Tbx21* locus. Th1-specific hyper-accessible regions are shaded with yellow. **d**. Boxplots of expression of Th1-specific hyper- or hypo- accessible regions nearest genes, values are based on log_2_(FPKM + 1). The boxplots are shown as median (line), interquartile range (box), and minimum to maximum data range (whisker). The p-values by paired Wilcoxon test, * < 0.05, ** < 0.01,*** < 0.001. **e** Correlation analysis of DARs and their nearest DEGs that were simultaneously up- or down-regulated in Th1. Each dot represents a gene that is significantly differentially expressed and associated with chromatin accessibilities changed compared to Naive (blue) or Th17 (orange). The Pearson correlation coefficient (r) and the corresponding P value are shown.

The large-scale changes in gene expression and chromatin accessibility during T cell differentiation are T cell-type specific^20^. Firstly, we specifically identified differentially expressed genes (DEGs) among three cell lines by comparing RNA-Seq data. The genes that were up- or down-regulated in expression in only one cell type were defined as cell-type specific DEGs. In general, 297, 392 and 479 up-regulated genes and 611, 198 and 444 down-regulated genes were identified in Naive, Th17 and Th1, respectively (Fig. 1a). Th17 up-regulated DEGs were involved in leukocyte mediated immunity, which was consistent with the fact that Th17 mediated immune responses against extracellular bacteria and molds by secreting interleukins. Correspondingly, Th1 up-regulated DEGs were enriched in the regulation of defense response and virus response (Supplementary Fig. 3c), which was consistent with the role of Th1 in the immune response against intracellular bacteria and protozoa. These results indicate that the cell-type specific DEGs caused by T cell differentiation play important roles in the immune response and specific physiological functions.

Then, we examined the changes of local chromatin accessibility and found that the peak density of accessible chromatin regions increased (Fig. 1b), which was consistent with previous report that T cell differentiation led to increased chromatin accessibility^20^. We defined the cell-type specific differentially accessible regions (DARs) including hyper- and hypo-accessible regions. In general, 4428, 6119 and 7123 hyper-accessible regions and 2449, 2617 and 2405 hypo-accessible regions were identified in Naive, Th17 and Th1 cells, respectively (Supplementary Fig. 3d). The functional enrichment analysis of cell-type specific hyper-accessible regions using GREAT^30^ revealed that Th17 specific hyper-DARs were associated with T cell differentiation, while Th1 specific hyper-DARs were enriched in the function of immune response (Supplementary Fig. 3c). When we annotated the cell-type specific hyper-accessible regions to the nearest transcription start site of genes by HOMER^31^, these open chromatin sites were mainly located in the vicinity of functional genes. For example, Naive cells displayed hyper-accessibilities near Kruppel-like factor 4 (*Klf4*) locus which was highly expressed in Naive T cells but down-regulated upon T cell activation^2^. Th17 cells showed increased accessibilities around RAR-related orphan receptor gamma (*Rorc*) locus which had critical functions for Th17 differentiation (Supplementary Fig. 3e). Similarly, Th1 cells showed increased accessibilities near the T-box 21 (*Tbx21*, known as T-bet) locus which was lineage-determining factor for Th1 (Fig. 1c). This is in line with previous studies^20^ that T cell differentiation leads to lineage-specific changes in local chromatin accessibility.

To examine the potential relationship between DARs and DEGs, we performed statistical analysis of the expression of genes nearest cell-type specific DARs and focused on the correlation of simultaneously increased and decreased DARs and DEGs in a cell-type specific manner in three kinds of T cell. Consistent with previous studies^32^, we found that the genes in open chromatin regions tended to have higher expression levels (Fig. 1d and Supplementary Fig. 3f), and the changes in cell-type specific DARs and DEGs were positively correlated (Fig. 1e and Supplementary Fig. 3g).

### Global alterations in *cis* chromatin interactions during T cell differentiation

**Fig. 2:**
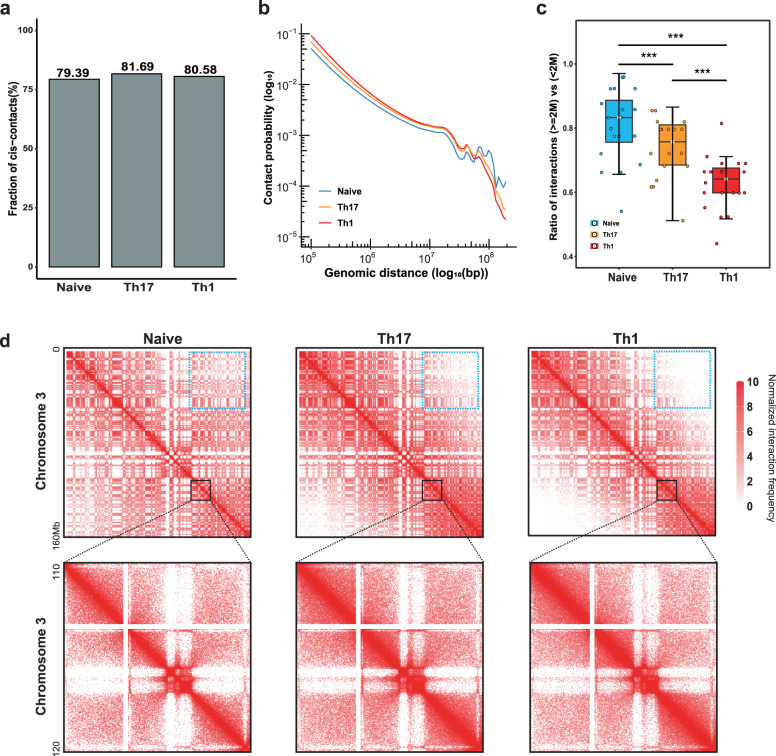
T cell differentiation leads to increased short-range and decreased extra-long-range chromatin interactions. **a** Fraction of *cis* chromatin contacts. **b** The chromatin contact probabilities (*P(s)*) relative to genomic distance for Naive, Th17 and Th1, respectively. **c** Boxplot of interactions ratio of (>= 2 Mb)/(< 2 Mb). The boxplots are shown as median (line), interquartile range (box), and minimum to maximum data range (whisker). The p-values by paired Wilcoxon test, *< 0.05, **< 0.01, ***< 0.001. **d** Hi-C produces a genome-wide contact matrix. The normalized Hi-C interaction frequencies (100-kb bin, chromosome 3) in each sample. Zoomed-in views (40-kb bin) are also shown. Each pixel represents all interactions between a 100-kb (or 40-kb) locus and another 100-kb (or 40-kb) locus; intensity corresponds to the ICE normalized values (0–10); the blue dotted box indicates extra-long-range (> 80 Mb) regions.

To explore the dynamics of the three-dimensional chromatin structure during T helper cell differentiation. We first examined the proportion of *cis* chromatin interactions in non-activated and activated T cells. The result showed that the total *cis* interactions were markedly increased in the activated T cells (Fig. 2a and Supplementary Fig. 4a). To scrutinize the distribution of intra-chromosomal contacts in detail and preclude the distance effect, we performed the Relative Contact Probability analysis described by Lieberman-Aiden et al.^33^ to compute the contact probability as a function of genomic distance (*P(s)*). For murine T cells, short-range (<2 Mb) and intermediate-range (2–10 Mb) interactions were increased in Th cells, while the curve steeply declined once the genomic distance exceeded ~80 Mb (extra-long-range interactions) (Fig. 2b). Similarly, the activated cells had more short-range interactions and fewer long-range interactions than the resting in human T cells, with a rapid drop-off at ~12 Mb (Supplementary Fig. 4b), which was consistent with the findings during B cell activation^34^. To better capture short-range interactions, we then calculated the ratio of normalized interaction frequencies over genomic distance greater and less than 2 Mb for each chromosome and discovered that the ratio was significantly lower in Th cells (Fig. 2c). When the cells were activated, the typical checkerboard pattern of compartments visually shrunk away from the diagonal, indicating increased interaction frequencies at short-range distance scales and decreased interaction frequencies at extra-long-range distance scales (Fig. 2d and Supplementary Fig. 4c, d). In overall, T cell activation and differentiation results in globally increased short-range and decreased long-range *cis* interactions.

### Weaker compartmentalization in T helper cells

**Fig. 3:**
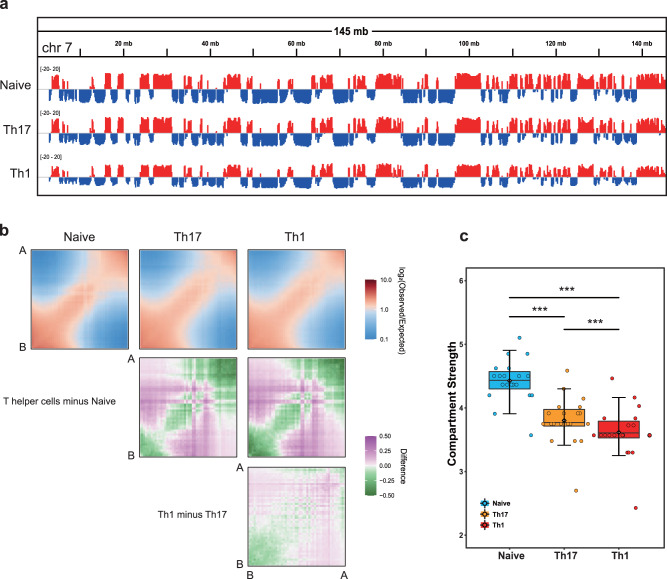
Differences in compartmentalization. **a** The first principal component (PC1) values calculated from the Hi-C data (100-kb bin) in Naive, Th17 and Th1 cells along chromosome 7 indicate the stability of the A/B compartments. **b** Saddle-plots and differential saddles are displayed. Bins are assigned to fifty deciles based on PC1 values, average observed/expected distance-normalized scores for each pair of deciles are simultaneously calculated. **c** Boxplot of the compartmentalization-strength per chromosome-arm (dots). The boxplots are shown as median (line), interquartile range (box), and minimum to maximum data range (whisker). The p-values by paired *t*-test, *< 0.05, **< 0.01, ***< 0.001.

To further investigate the chromatin structure dynamics, we next examined the compartment level of T cells. It is well established that rearrangement of chromosome A/B compartments is influenced by lineage differentiation^35^. In each cell line, the genome was divided into about 50% A and 50% B compartments (Supplementary Fig. 5a). When naive cells were differentiated into various Th cells, we only discovered a small proportion of compartments that were switched (Fig. 3a and Supplementary Fig. 5b). A-compartments were typically associated with open chromatin, whereas B-compartments were associated with closed chromatin^33^. Consistent with previous reports^21,35^, the proportion of regions switched from A to B had lower gene expression, whereas regions switched from B to A had higher gene expression (Supplementary Fig. 5c). This subtle switchable pattern suggests that T cell differentiation has modest effect on the A/B compartment switch. Then, we investigated *cis*-interactions between A- and B- compartments to interpret changes in compartmentalization. When compared to naive cells, intra-compartment interactions (A-A or B-B, *cis*) decreased while inter-compartment interactions (A-B, *trans*) increased. Furthermore, Th17 had more intra-compartment interactions and fewer inter-compartmental interactions than Th1 (Fig. 3b). This phenomenon could be further investigated using compartment strength, which refers to the proportion of intra- versus inter-compartment interactions calculated separately for each chromosome^36,37^. In comparison to Naive cells, Th17 and Th1 showed the reduced compartmentalization (Fig. 3c). Similarly, we also found that compartmentalization strength was weakened during human T cell activation (Supplementary Fig. 5d, e). These findings indicate that while T cell activation and differentiation do not extensively convert A/B compartments, they do lead to the reduced compartmentalization strength in activated cells compared to non-activated cells.

### T cell differentiation induces TAD rearrangement

**Fig. 4:**
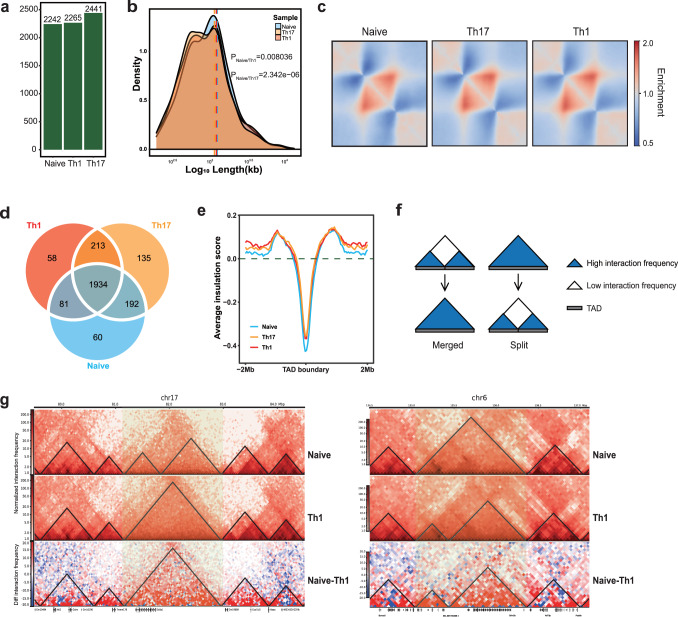
T cell differentiation results in TAD rearrangement. **a** TAD number of each sample (pooled data from two biological replicates of each sample). **b** Density plot shows TAD length distribution. The dotted lines represent the mean TAD length, *p* values are calculated by unpaired Wilcoxon test. **c** Rescaled observed/expected pileups of TADs at 40-kb resolution. **d**. Overlap of TAD boundaries. **e** The average insulation scores around TAD boundaries (± 2 Mb). **f** Schematic depicts TAD and TAD boundaries alteration events. **g** Heatmap of the Merged and Split events between Naive and Th1. The yellow box indicates the area where the event occurs.

We observed the increase of short-range interactions, which could be attributed to the gain of TADs^38–40^. Therefore, we then checked the structural change on TAD level. Upon T cell activation, TADs appeared to be increased in number but smaller in size than those in non-activated T cells (Fig. 4a, b and Supplementary Fig. 6a, b), which was consistent with the previous report^21^. Since TADs had different sizes, we thus performed the Aggregate TAD Analysis (ATA) to rescale them to a uniform size to compare intra-TAD contacts across different T cells. The ATA analysis revealed the increased intra-TAD contacts in activated T cells (Fig. 4c and Supplementary Fig. 6c). In Naive, Th17 and Th1 cells, we identified 2266, 2473 and 2285 TAD boundaries, respectively and most boundaries were conserved across cell types (Fig. 4d and Supplementary Fig. 6d). According to the insulation score^40^ analysis which can reflect boundary intensity, boundary insulation was weakened in activated T cells (Fig. 4e and Supplementary Fig. 6e). Together, these observations imply TADs undergo global changes upon T cell activation.

Previous studies revealed the Merged and Split events are the most disruptive changes in 3D structure^24,41,42^. Merged represents two smaller TADs in Naive fused into one larger TAD in Th cells, while Split is the inverse event of Merged (Fig. 4f, g and Supplementary Fig. 6f). TADs are conserved across cell types and species^38,43^. We found that Th17 and Th1 had 80 and 79 Merged and Split events in common, respectively (Supplementary Fig. 6g, h). It prompted us to investigate whether TADs were also homologous in mouse and human activated T cells. Indeed, higher-order structure in mouse and human syntenic regions was quite similar (Supplementary Fig. 7), implying that genome structure in T cell activation was conserved beyond primary sequences in DNA. Taken together, these results suggest that T cell activation and differentiation lead to TAD rearrangement which is conserved across Th cells and species.

### Chromatin interactions in response to T cell differentiation

**Fig. 5:**
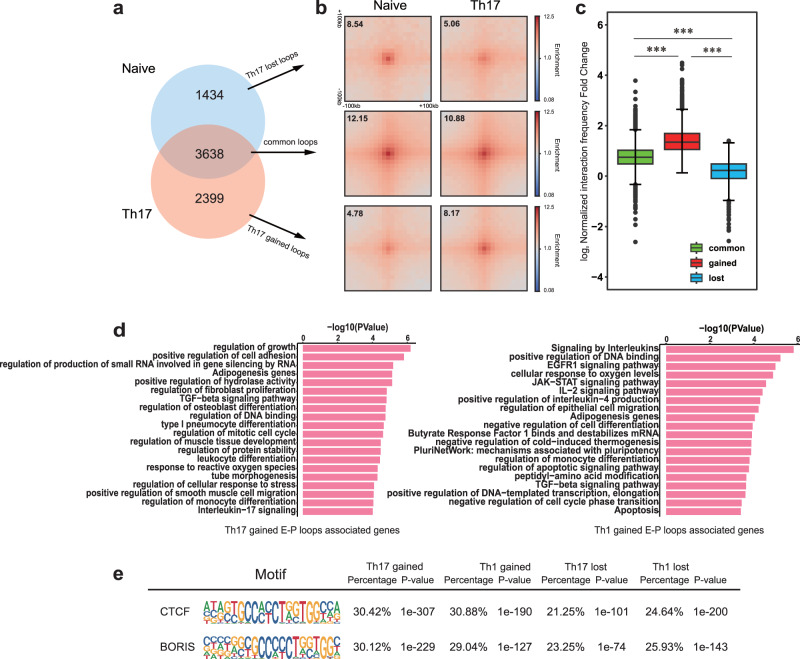
T cell differentiation brings about genome-wide changes in chromatin interactions. **a** Venn diagram shows number of loops in Naive and Th17 cells. **b** Aggregate peak analysis (APA) at 10-kb resolution plots display the average Hi-C signal at chromatin loops that are lost, gained, and common during differentiation from Naive to Th17. The enrichment value in the central pixel is displayed on top left for reference. **c** The boxplot shows the log_2_ normalized interaction frequency fold change (Th17/Naive) for the three groups in **a**. The boxplots are shown as median (line), interquartile range (box), and minimum to maximum data range (whisker). The p-values by unpaired Wilcoxon test, ***< 0.001. **d** Enrichment analysis of genes associated with E-P gained loops. **e**. The top two motifs within ATAC-Seq peaks associated with E-P loops.

High-order chromatin structure plays important roles in gene regulation^44,45^. Gene transcription could be modulated by regulatory elements located in the loop anchors, such as typical enhancer clusters. And loops between promoters and enhancers are strongly associated with gene activation^46^. Therefore, we identified loops at 10-kb and 25-kb resolutions using HiCCUPS^46^ to connect changes in 3D genome structure with gene regulation. We identified 5072, 6037 and 4189 loops in Naive, Th17 and Th1 cells, respectively. Loops across cell lines were of high conservation^46^. In contrast to loops in naive cells, the loops in the two Th cells showed more similarity (Fig. 5a and Supplementary Fig. 8a). We further divided loops into Gained, Lost and Common for T helper cells. And the Aggregate Peak Analysis provided a characterization of three groups of loop density enrichment by aggregating small portions of the Hi-C matrix from a two-dimensional set of locations to get a genome-wide impression^43,46^. The intensity of interaction frequency between groups showed significant differences (Fig. 5b, c and Supplementary Fig. 8b, c).

To detect cell-type specific E-P loops, we took advantage of ATAC-Seq data to divide the accessible chromatin regions into promoter and non-promoter (the putative enhancer clusters) regions. We then looked at the Th-specific E-P loops and performed enrichment analysis of genes associated with them. The genes associated with Th17-gained E-P loops were enriched in leukocyte differentiation and interleukin-17 signaling, while genes associated with E-P loops acquired by Th1 were involved in signaling by interleukins and cellular response to oxygen levels (Fig. 5d). Both Th17- and Th1-lost E-P loops associated genes participated in T cell receptor (TCR) signaling pathway (Supplementary Fig. 8d). These results indicate that T helper cells with a variety of physiological functions could regulate gene expression via chromatin interactions and genes associated with Th-specific E-P loops are relevant with activation of certain specific genes in T helper cells.

CCCTC-binding factor (CTCF) is essential to define TAD boundaries and to facilitate the formation of insulated chromatin loop structures^47^. Previous research has found that CTCF binding at loop anchors can connect enhancers and promoters and positively correlate with gene activation in Th2 cells^23^. We discovered that CTCF binding sites were enriched around loops we identified (Supplementary Fig. 8e). To investigate the role of CTCF in the formation of Th cell-type specific E-P loops, we measured CTCF enrichment adjacent to loop anchors, and found that CTCF densities associated with gained- and lost-loops differed. (Supplementary Fig. 8f). Additionally, both gained- and lost- loops were associated with CTCF and BORIS binding motifs (Fig. 5e). These findings suggest that CTCF binding sites may be involved in establishing chromatin loops during T cell differentiation.

### Cell-type specific enhancer-promoter loops in T helper cells

**Fig. 6:**
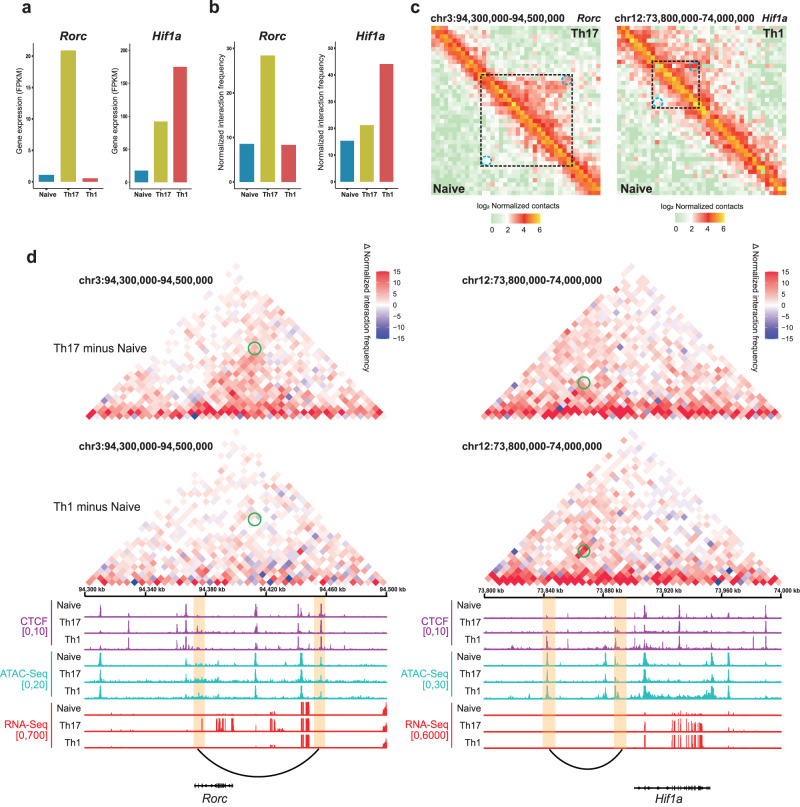
Cell-type specific gene-regulatory interactions of T helper cells. **a** The gene expression (FPKM) of *Rorc* and *Hif1α*, respectively. **b** The normalized interaction frequencies of *Rorc* and *Hif1α*, respectively. **c** The non-subtraction Hi-C maps of *Rorc* and *Hif1α* locus, respectively. The dashed boxes indicate the loop range. The blue dashed circles indicate the Th-specific E-P interaction. **d** The heatmap shows the differential normalized interaction frequencies at the *Rorc* / *Hif1α* locus between Th17 and Naive, between Th1 and Naive. The green circles indicate the E-P interaction in Th17/Th1 but not in Naive. The bottom tracks show CTCF signals, ATAC-Seq signals, gene expression and Refseq gene. Regions of E-P loop anchors are shown in yellow boxes.

Enhancers and promoters can be located on both sides of the loop and promote gene expression^46^. Therefore, we continue to examine the role of Th-specific E-P interactions whose changes on CTCF binding or distal enhancers occurred around loop anchors. In particular, we found that the promoter of *Rorc* was bound by CTCF only in Th17 cells and interacted with enhancer clusters ~80-kb downstream to form a chromatin interaction in Th17 but absent in Naive and Th1 cells (Fig. 6a–d). RORγt has been identified as a lineage-specific transcription factor that promotes Th17 cell differentiation and proliferation. The dynamic regulation of RORγt expression, stability, and activity determines Th17 function^9^. c-Maf increases Il4 expression in Th2 cells and influences Th17 and Tfh cell differentiation^48^. A Th17-specific E-P loop was also detected at *Maf* locus (Supplementary Fig. 9a-d) which connected the upstream Th17 specific enhancer in Th17 but not in Th1 cells. HIF-1α (hypoxia-inducible factor 1α) is a Th1 determinant whose activity promotes glycolysis under hypoxia^49^. The upstream enhancer formed a ~ 50-kb E-P interaction with the promoter of *Hif1a*. CTCF binding was observed at both anchors of this loop only in Th1 cells (Fig. 6a–d). Compared to Naive and Th17 cells, the interaction frequencies and expression of *Hif1a* locus in Th1 were also increased (Fig. 6a, b). Another Th1-specific E-P loop was discovered at the *Jun* locus who was one of the activating protein-1 (AP-1) transcription complexes and the most active TF in the AP-1 complex as well^50^ (Supplementary Fig. 9a–d). These findings imply that numerous genes could form CTCF-bridged chromatin interactions at their promoters and distal enhancers to regulate gene expression during T cell differentiation.

## Discussion

Cell differentiation critically depend on precise temporal-spatial control of transcription programs. Mammalian cell differentiation also accompanies the changes in chromatin structure^35^. During human cardio genesis, dynamics of genome reorganization reveal an RBM20-dependent regulation network^51^. Disruption of TADs can rewire long-range regulatory landscape and cause pathogenic phenotypes, such as one that different limb pathogenic structural variations are associated with disruptions of TAD structure at the EPHA4 locus in human and mouse^44^. Although chromatin higher-order architecture of T cells has been studied^21^, it remains unclear how chromatin structure changes after non-activated Naive CD4^+^ T cells differentiation into various T helper subsets. In the present study, we used multi-omics data to characterize multi-level synergistic changes during T cell differentiation.

The mechanisms of CD4^+^ T cell differentiation in terms of definitive cytokines, master transcriptional regulators, and signaling pathways are similar in human and mouse Th cell subsets, indicating T cell differentiation is quite conserved across species^48^. Multiomics assays are executed on mouse Naive CD4^+^ T cells that differentiate in vitro to study epigenome dynamics during differentiation, which has been the subject of numerous prior reports^25–28^. Based on the integrated analysis of DEGs and DARs, transcriptome expression and chromatin accessibility are significantly correlated at lineage-determining genes like *Rorc* for Th17 and *Tbx21* for Th1. However, comprehensive studies on comparison of the chromatin higher-order structure of human and mouse during T cell activation are rarely mentioned. We find that changes in chromatin higher-order structure during activation are quite conserved across Th cells and species, including increased *cis* chromatin contacts, increased short-range and decreased long-range interactions, weaker compartmentalization and gain of intra-TAD interactions. These findings shed light on the general patterns of 3D genome changes that occur during T cell activation in human and mouse.

The regulatory mechanisms of T cells are complicated and diverse, which is susceptible to the changing microenvironment and surrounding molecules. T helper cell lineage commitment was originally thought to be a one-way process, namely irreversible terminal differentiation of Th1 and Th2 cells. However, recent studies have shown that Th1 can convert to Th2 under certain conditions^52,53^. In addition, Th17 cells have been reported to be more plastic than Th1/Th2 cells^53,54^. The plastic phenotypes of Th17 cells have important biological implications for immune intervention^4^, while the underlying molecular mechanisms are poorly understood. One outstanding study points out that metabolic heterogeneity underlies Th17 cell stemness and plasticity^55^. According to our findings on high-order chromatin structure across three types of T cell, for instance, changes in *cis* chromatin contacts (Fig. 2b–d) and compartmentalization difference (Fig. 3b, c), the cell plasticity of different T helper cells appeared to be reflected by these structural changes. For example, Th1 cells showed more increased short-range interaction and further reduced compartmentalization than Th17 cells during differentiation, consistent with the fact that Th1 cells are more differentiated cell type than Th17 cells. To some extent, we speculate that the 3D chromatin structure of other cell types such as embryonic stem cells and tissue stem cells may also be associated with their cell plasticity, which requires analyses of multi-omics data and rigorous experiments to explore and verify .

The marked changes of insulation at TAD boundaries may not have huge direct impacts on the transcription program. The extreme case of the change of TAD insulation has been reported by Nora, E. P. et al. ^56^, in which the acute depletion of CTCF leads to dramatic loss of TAD insulation but only has modest impacts on gene transcription. Considering the change of TAD insulations, we have observed in T cell differentiation is much weaker than the change caused by CTCF depletion, it is difficult to directly link the change of gene transcription to the change of TAD insulation. Therefore, it needs more sophisticated experimental designs to explore the relationship between change of TAD insulation and change of gene expression. A classic model in which loop anchors between a promoter and enhancer activate a target gene is widely recognized^46,57^. We identified many cell-type specific E-P loops in Th cells. Previous studies have shown that the conserved non-coding sequences control cytokine-induced *Rorc* transcription during Th17 differentiation^10^. Interestingly, we found a ~ 80 kb Th17-specific loop at the *Rorc* locus. This is an important supplement to study the relevant mechanisms of Th17. Nonetheless, no cell-type specific E-P loops were discovered at the T-bet locus, an important transcription factor for Th1 differentiation^4,58^, indicating that not all Th cell differentiation related genes are activated in the E-P interaction manner. These cell-type specific E-P loops, which are a manifestation of chromatin organization heterogeneity, are epigenetic landscape units in T helper cells. Perhaps it is caused by an increase in the frequency of chromatin interactions and corresponding sites maintaining or obtaining CTCF binding and ATAC signal, which requires further study. Future research is also needed to determine whether these interactions contribute to T cell differentiation and function. In order to better capture the general patterns of changes on 3D genome organization during T cell differentiation between humans and mice, it requires multiple omics resources of human naive CD4^+^ T cells and various T helper cell subsets. Still, ground on our bioinformatic analysis, these data provide a comprehensive reference for scientists in the related fields.

The diversity of T cell differentiation is not only reflected in the transcriptome and chromatin accessibility level, but also in the higher-order chromatin structure. Once Th17 converts to Th1, it is unclear whether the 3D genome organization will change synchronously, whether Th17-related loops will be lost, and whether Th1-related loops will appear. How the landscapes of chromatin structure in other T helper subsets like Th2, Treg and Tfh change upon differentiation needs further investigation. In summary, we used RNA-Seq, ATAC-Seq, and Hi-C to characterize mouse Naive CD4^+^ T cells differentiate into Th17 and Th1 cells, demonstrating the dynamic changes of multi-scale 3D genome organizations associated with cell-type specific gene regulation during T cell differentiation. This study links chromatin structure characteristics to gene expression, providing a meaningful epigenetic landscape for T cell differentiation from the perspective of chromatin higher-order architecture.

## Methods

### Naive CD4^+^ T cell isolation and cell preparation

C57BL/6 mice (1-2 months old) were purchased from Slac Laboratory Animals Inc. Miltenyi Biotec’s isolation kit for mouse naive CD4^+^ T cells (CD4^+^CD62L^+^ T Cell Isolation Kit II, mouse, #130-093-227) was used to separate CD4^+^CD44^-^ naive T cells from pooled lymph nodes and spleens. FACS determined 95% purity of the isolated CD4^+^ T cells. Cells were cultured in complete RPMI 1640 medium (Corning) supplemented with 10% FBS (Invitrogen), 200 mM glutamine (Gibco), 100 mM sodium pyruvate (Gibco), 50 μM β-mercaptoethanol (Gibco), 100U/ml penicilin and 100 μg/ml streptomycin. For in vitro induction, CD4^+^CD44^-^ naive T cells (1×10^6^/well) were cultured in 48-well flat-bottom tissue culture plates (Corning Costar) with 1 μg/ml-bound of anti-CD3 (BD, #553057) and 3 μg/ml of soluble anti-CD28 (BD, #55329) or activated by Dynabeads® Mouse T-Activator CD3/CD28 (Life Technologies, #11452D) in culture medium in the presence of antibody and cytokines: for Th1 conditions, anti-IL-4 (10 μg/ml) (BD, #554432), IL-12 (20 ng/ml) (R&D, #419-ML) and IL-2 (10 ng/ml) (Peprotech, #200-02); for Th17 conditions, anti-IL-4 (10 μg/ml), anti-IFN-γ (10 μg/ml) (BD, #554408) and TGF-β (1 ng/ml) (Peprotech, #AF-100-21C), IL-6 (100 ng/ml) (Peprotech, #216-16), IL-1β (10 ng/ml) (Peprotech, #211-11B) and IL-23 (20 ng/ml) (R&D, #1887-ML); At the 5th days of culture, Th cells were collected for RNA-Seq, ATAC-Seq and Hi-C assays or for cellular cytokine staining and flow cytometry. All animal experiments were performed in accordance with protocols approved by the Shanghai Institute of Nutrition and Health Institutional Animal Care and Use Committee.

### Surface and intracellular staining for FACS

For surface staining of CD4 cells, cells were surface stained for 15–30 min at 4 °C with anti-mouse CD4-FITC (eBioscience, #11-0041), anti-human/mouse CD44-PE (eBioscience, #12-0441) and anti-mouse CD62L-APC (eBioscience, #17-0621) in PBS supplemented with 2% FBS, the cells were washed and collected for analysis on BD Accuri C6 Flow cytometer, data were analyzed with CFlow Plus software. For intracellular staining of specific cytokines in Th cells, cells were stimulated at 37°C for 4 h with 50 ng/ml PMA (MultiSciences Biotech, #CS0001) and 750 ng/ml ionomycin (MultiSciences Biotech, #CS0002) and Golgistop was added to block cytokine secretion (BD, #554724). After stimulation, cells were fixed and permeabilized with Cytofix/Cytoperm (BD, #554722) for 20 min on ice and then stained with anti-mouse IFN-FITC (eBioscience, #11–7311), anti-mouse IL17A-Alexa Fluor^®^ 647 (Biolegend, #506911) and anti-mouse IL-4-PE (eBioscience, #12–7041). Intracellular staining of Foxp3 was performed according to the instruction of Foxp3 staining kit from eBioscience (Anti-mouse/rat Foxp3 set PE, #72–5775). Samples were acquired on a BD FACS Celesta flow cytometer and data analysis was conducted using FlowJo software. med according to the instruction of Foxp3 staining kit from eBioscience (Anti-mouse/rat Foxp3 set PE, #72–5775). Samples were acquired on a BD FACS Celesta flow cytometer and data analysis was conducted using FlowJo software.

### RNA-Seq library construction and sequencing

Total RNA was isolated using the RNeasy Plus Mini Kit from Qiagen (#74134), and concentrations and integrity were determined using an Agilent Bioanalyzer. The RNA-seq library was conducted by Novogene Company and sequenced on an Illumina HiSeq X Ten sequencer with pair-end 150 bp reads.

### ATAC-Seq library construction and sequencing

To prepare the nuclei, 25,000–50,000 Th cells were lysed in lysis buffer (10 mM Tris-HCl (pH 7.4), 10 mM NaCl, 3 mM MgCl_2_ and NP-40) for 10 min on ice. For all Th cell types, the optimal NP-40 concentration is 0.5%. The nuclei were spun at 500 g for 5 min immediately after lysis to remove the supernatant. The nuclei were then incubated for 30 min at 37 °C with the Tn5 transposome and tagmentation buffer (Vazyme Biotech). To end the tagmentation, the stop buffer was directly added into the reaction after the tagmentation. PCR was used to amplify the library for 13 cycles at 72 °C for 3 min, 98 °C for 30 s, and thermocycling at 98 °C for 15 s, 60 °C for 30 s, and 72 °C for 3 min, followed by 72 °C 5 min. Following the PCR reaction, libraries were purified using 1.2X AMPurebeads (Beckman, #A63880). On an Illumina HiSeq X Ten sequencer, the final library was sequenced with pair-end 150 bp reads.

### In situ Hi-C protocol

Ten million cells were cross-linked with 1% formaldehyde for 10 min at room temperature. Nuclei were permeabilized. DNA was digested with DpnII (NEB), and the ends of restriction fragments were labeled using biotin-14-dATP (Life Technologies) and then ligated in a small volume. After reversal of crosslinks, ligated DNA was purified and sheared to a length of roughly 400 base pairs by sonication (Covaris S220), at which point ligation junctions were pulled down with MyOne Streptavidin C1 Dynabeads (Life Technologies) and prepared for Illumina sequencing. Isolated DNA was end-repaired before dATP-tailing with Klenow exo-(NEB), and they were ligated to Illumina paired-end sequencing adapters. Bead-bound Hi-C DNA was amplified with 11–13 rounds of PCR amplification. The final library was sequenced on an Illumina HiSeq X Ten sequencer with pair-end 150 bp reads.

### RNA-Seq data processing

RNA-Seq reads were mapped to the mm10 reference genome using Hisat2 (v2.2.1)^29^ with the parameters '–dta–no-discordant–no-mixed'. Duplicates were removed, and aligned reads were calculated for each protein-coding gene using HTSeq (v0.13.5)^59^ with default parameters. Fragments Per Kilobase of exon model per Million mapped fragments (FPKM) was calculated by StringTie (v2.1.7)^60^ with the default parameters. DESeq2 (v1.34.0)^61^ was applied to identify the differentially expressed genes with adjusted *p* value < 0.01 and |log_2_(FoldChange)|>1. Gene ontology was conducted with Metascape^62^. Gene tracks normalized to reads per million with Deeptools (v3.5.1)^63^ were generated in IGV^64^.

### ATAC-Seq data processing and correlation analysis of DARs and DEGs

ATAC-Seq reads were mapped to the mm10 with Bowtie2 (v2.3.5.1)^65^ using parameter '-X 2000–no-discordant–no-mixed–no-unal'. Aligned reads were filtered for a minimum MAPQ of 30, and duplicates were removed using SAMtools (v1.3.1)^66^. MACS2^67^ was applied to call peaks. The bigwig files were generated by Deeptools using RPKM option of bamCoverage command. To eliminate the bias across cell lines, we performed the quantile normalization of samples. Differentially accessible peaks were obtained by clustering normalized peak density Z-score. Gene ontology analysis of associated peaks was performed by online GREAT^30^ tool. Identification of nearest genes of DARs was performed using the annotatePeaks.pl script in HOMER^31^. The DARs nearest genes (filter by distance to transcription start site < 100 kb) were then overlapped with DEGs identified from RNA-seq. Fold changes of DARs and their nearest DEGs were used to calculate the Pearson correlation coefficient (PCC) and P-value in R.

### ChIP-Seq data processing

Reads were downloaded from GEO for the corresponding samples: CTCF Naive CD4^+^ T cells (GSM3498282), CTCF Th17 cells (GSM3498288)^68^ and CTCF Th1 cells (GSM1480825)^25^. Single-end reads were mapped to mm10 with Bowtie2. Peaks were called by MACS2 callpeak command. Genome coverage was calculated with Bedtools genomeCoverageBed program with an extension of 200 bp. The bigwig files were generated by Deeptools bamCoverage command.

### Hi-C sequence data processing

Paired-end sequencing reads were mapped to mm10 using HiC-Pro (v2.11.4)^69^ pipeline. The raw contact matrices were generated for each replicate at binning resolutions of 5-kb, 10-kb, 20-kb, 25-kb, 40-kb and 100-kb. To correct bias, the raw contact matrices were normalized using the iterative correction and eigenvector decomposition (ICE) method^37^. Heatmaps for Hi-C data were generated through R or pyGenomeTracks^70^. To generate the maximum counts of reads, we pooled two biological replicates for each cell line for subsequent analyses.

### Relative Contact Probability analysis

The raw contact sparse matrix with 100-kb resolution was applied to calculate the contact probability (*P(s)*) relative to the genomic distance. We counted the interactions in each bin and calculate *P(s)* by dividing the number of interactions in each bin by the total number of interactions across all bins.

### Analysis of A/B compartments

Calling A/B compartments by performing principal component analysis on distance-corrected, ICE normalized Hi-C matrices at 100-kb resolution. The principal component which correlated well in absolute value with corresponding ATAC-Seq signal called as above was chosen as representative of A/B compartments. The sign of the A/B compartments vector was set to match the sign of the correlation with ATAC-Seq signal, depicting that the positive values represented A compartment regions and negative values represented B compartment regions. Saddle plots were generated by GENOVA^71^ to assign each bin to its corresponding percentile value and dividing the genome into fifty sets of deciles. Measuring the compartmentalization strength to compare the average observed/expected values between inter- and intra- compartment, which can be thought of taking the ratio as follow:

$${{\mbox{Compartmentalization}}}\,{{\mbox{Strength}}}=\frac{{{\mbox{AA}}}\times {{\mbox{BB}}}}{{{{\mbox{AB}}}}^{2}}$$

### TAD analysis

Using directionality index^38^ profile at 40-kb resolution to perform hierarchical clustering at TAD level. Insulation score^40^ was calculated by matrix2insulation.pl public script with '–is 1e6–ids 2e5–nt 0.25' options, using ICE normalized Hi-C matrices at 40-kb resolution. Conserved boundaries were defined as if there was a boundary within ± 80-kb compared to other cells, cell-type specific boundaries were identified by the lack of shared boundaries with the respective comparison. TADs were identified using hicFindTADs command in HiCExplorer (v3.7.2)^70^. TADs can be investigated globally by aggregating Hi-C matrix around TADs. Using coolpup.py (v0.8.7)^72^ to perform aggregate TAD analyses, because TADs have different sizes, they are rescaled to a uniform size and then the result is averaged across the genome. The merged or split event regions were obtained by using Bedtools. Boundaries across species were carried out by hg38 lifting over to mm10 using UCSC liftover.

### Loop detection and definition of conserved and Th-specific E-P loops

We detected loops using HiCCUPS^46^ at 10-kb and 25-kb resolutions with default parameters for each cell line. Briefly, fold change values were calculated by quantifying enrichment of interaction frequency compared to local background. Cross-cell loop comparison was achieved and slightly modified by the Luo, X. et al.^43^. The distance judgement determines whether the mapped loop was located near any loop of target cell line. We defined a loop to be Th-specific compared to Naive if one of the two anchors extending after the resolution-length (10-kb or 25-kb, it depended on the resolution of the loop calling) did not intersect. Before we determined the E-P loops, we defined the ATAC-Seq peaks that overlapped with gene promoters (from -2-kb to +2-kb of transcription start site) as promoter regions, while the non-promoter regions of ATAC-Seq peaks were regarded as the putative enhancers. Then taking the intersection of these Th-specific loops with promoter of genes and ATAC-Seq non-promoter regions to acquire Th-specific E-P loops whose one anchor overlapped with promoters and the other anchor overlapped with the putative enhancers. For Th-specific E-P loops, we mainly paid attention to those with CTCF binding or enhancer changes at anchors. The Th-specific E-P loops associated genes were subjected to enrichment analysis performed by Metascape, and the putative enhancer regions that overlapped with Th-specific loop anchors were performed motif analysis by HOMER.

### Aggregated peak analysis

We performed Aggregated peak analysis using coolpup.py at ICE normalized 10-kb resolution and 100-kb window and exclude the first two diagonals. The APA matrix was plotted as the heatmaps. Values of enrichment in top left corners of pileups were the enrichment of interactions in the center pixel of the matrix, after all described normalization procedures.

### Human CD4^+^ T cells source and data analysis

Data of human CD4^+^ T cells are from GSE126117^21^. As for the data analysis of human CD4^+^ T cells was used the similar parameters except hg38 genome build for mapping reads.

### Statistics and reproducibility

Standard statistical analyses were performed using R. The type of what statistical tests to use, and test results were described in the figure legend. No experimental samples were excluded from statistical analysis. We performed reproducible checks on Hi-C data using normalized interactions restricted in 5 Mb, HiCRep^73^ for reproducibility score, PC1 values at 100-kb resolution and directionality index profiles at 40-kb resolution. The normalized reads density of ATAC-Seq and normalized counts of RNA-Seq data are for principal component analysis and calculating Pearson correlation coefficient.

### Reporting summary

Further information on research design is available in the Nature Portfolio Reporting Summary linked to this article.

## Supplementary information

Supplementary Information

Description of Additional Supplementary Files

Supplementary Data 1

Reporting Summary

## Data Availability

Sequencing data of RNA-Seq, ATAC-Seq and Hi-C have been deposited in the Gene Expression Omnibus (GEO) under accession number GSE210419. CTCF ChIP-Seq data are downloaded from GEO for the corresponding samples: CTCF Naive CD4^+^ T cells (GSM3498282), CTCF Th17 cells (GSM3498288) and CTCF Th1 cells (GSM1480825). The Hi-C datasets used for human CD4^+^ T cell 3D genome analysis are from GSE126117. The source data for graphs in the manuscript can be found in Supplementary Data 1. Any other relevant data or information about this study are available from the corresponding author upon reasonable request.

## Code availability

The codes for RNA-Seq data processing are included in the supplementary Codes and all other codes necessary to replicate the analyses are available upon request.
