# Supplementary material for: Multi-omic analysis reveals dynamic changes of three-dimensional chromatin architecture during T cell differentiation
Source: Commun Biol. 2023 Jul 24;6:773. doi: 10.1038/s42003-023-05141-1 (PMC10366224; doi:10.1038/s42003-023-05141-1)
Supplement: Supplementary file 1 — Supplementary Information [file 42003_2023_5141_MOESM1_ESM.pdf]

Supplementary Information

Multi-omic analysis reveals dynamic changes of three-dimensional chromatin architecture during T cell differentiation

Ge Zhang[1], Ying Li[1]* and Gang Wei[1]*

[1]CAS Key Laboratory of Computational Biology, Shanghai Institute of Nutrition and Health, University of Chinese Academy of Sciences, Chinese Academy of Sciences, Shanghai 200031, China

*Correspondence: liying2010@sibs.ac.cn; weigang@picb.ac.cn

Guide to content:

Supplementary Figures

Supplementary Fig. 1 Study overview and characterization of Naive CD4$^+$ and T helper cells by FACS

Supplementary Fig. 2 Reproducibility of Hi-C data.

Supplementary Fig. 3 T cell differentiation extensively influences gene expression and chromatin accessibility.

Supplementary Fig. 4 Similar trends in *cis* chromatin contact change in mouse and human T cell activation.

Supplementary Fig. 5 Analysis at compartment level.

Supplementary Fig. 6 TADs are changed by T cell differentiation.

Supplementary Fig. 7 TADs are conserved across T helper cells and species.

Supplementary Fig. 8 T cell differentiation brings about genomic-wide changes in chromatin interactions.

Supplementary Fig. 9 Epigenetic landscapes at *Maf* and *Jun* locus.

Supplementary Tables

Supplementary Table 1 RNA-Seq and ATACA-Seq data mapping information

Supplementary Table 2 Hi-C data mapping information

Other supplementary materials for this manuscript include the following:

Supplementary Codes

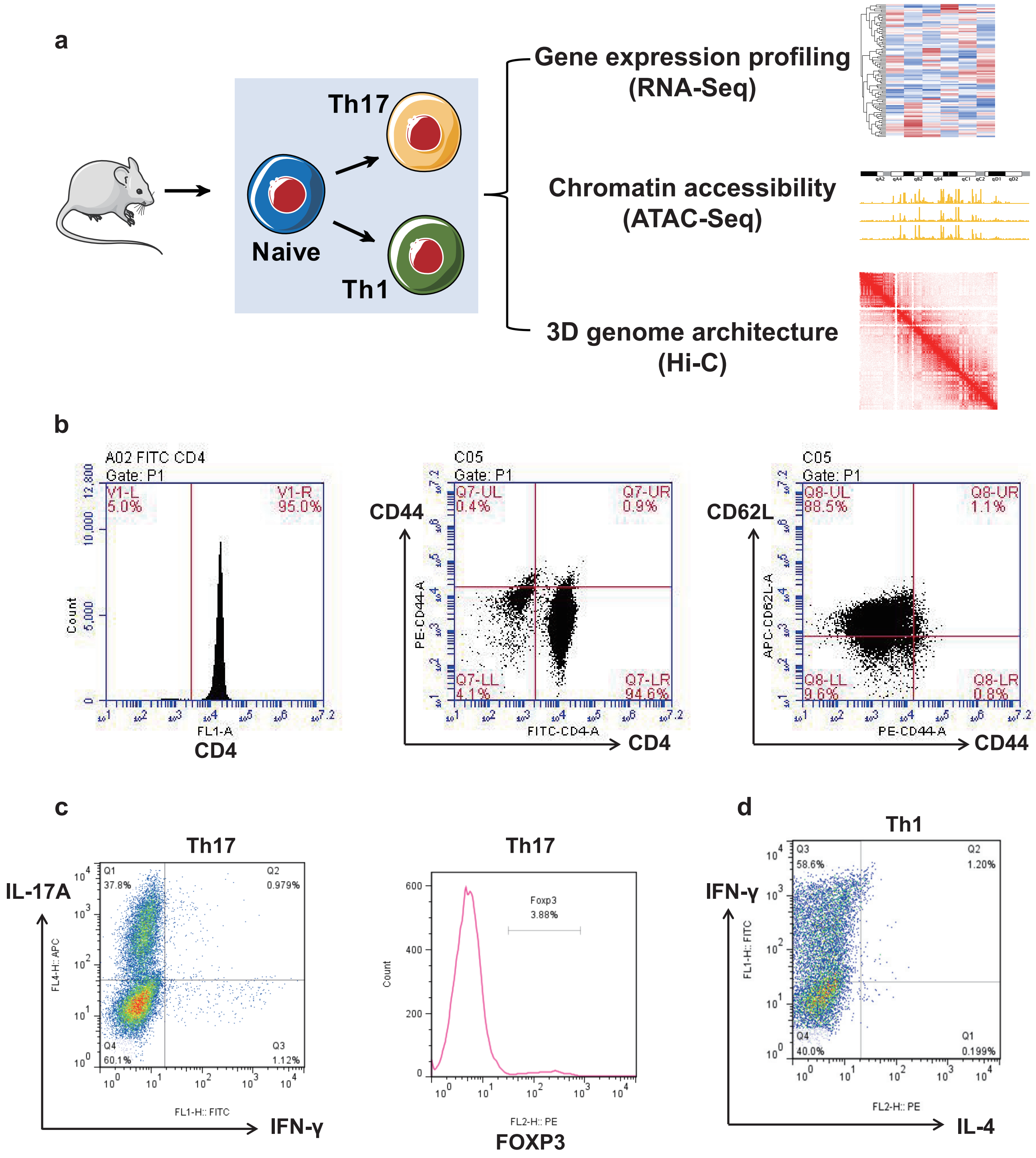

Supplementary Fig. 1 Study overview and characterization of Naive CD4$^+$ and T helper cells by FACS. a. Schematic of the study design. **b.** Naive CD4$^+$ T cells were isolated by MACS purification with CD4$^+$CD62L$^+$ T Cell Isolation Kit II, mouse (Miltenyi Biotec).Cell purity was assessed by staining for surface expression of CD4, CD44 and CD62L markers. **c.** For differentiated Th17 cells, the expression of IL-17A and IFN-γ were measured by intracellular staining. FOXP3 expression was also measured according to the manufacture's protocol of FOXP3 staining kit. **d.** For differentiated Th1 cells, the expression of IFN-γ and IL-4 were measured by intracellular staining.

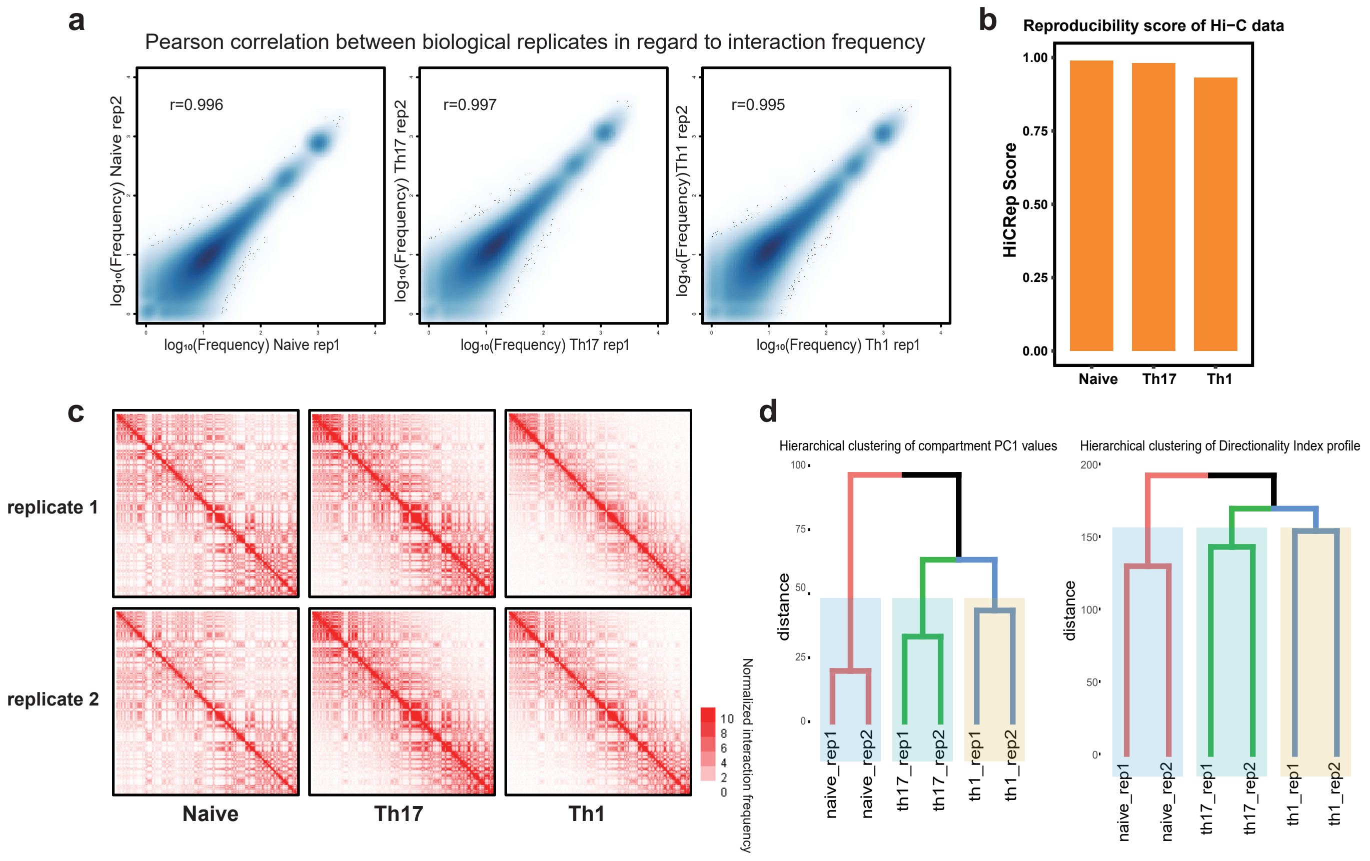

Supplementary Fig. 2 Reproducibility of Hi-C data. a. Density scatter plots for normalized interactions restricted in 5 Mb distance of biological replicates in 100-kb resolution. Pearson correlations are given. **b.** Reproducibility score of intra-chromosome interaction frequencies between the replicates at 40-kb resolution of Hi-C data by HiCRep method. **c.** Heatmap of normalized Hi-C interaction frequencies (100-kb bin, chromosome 1) in each replicate. **d.** Hierarchical clustering of compartment PC1 values at 100-kb resolution and directionality index (DI) profiles at 40-kb resolution.

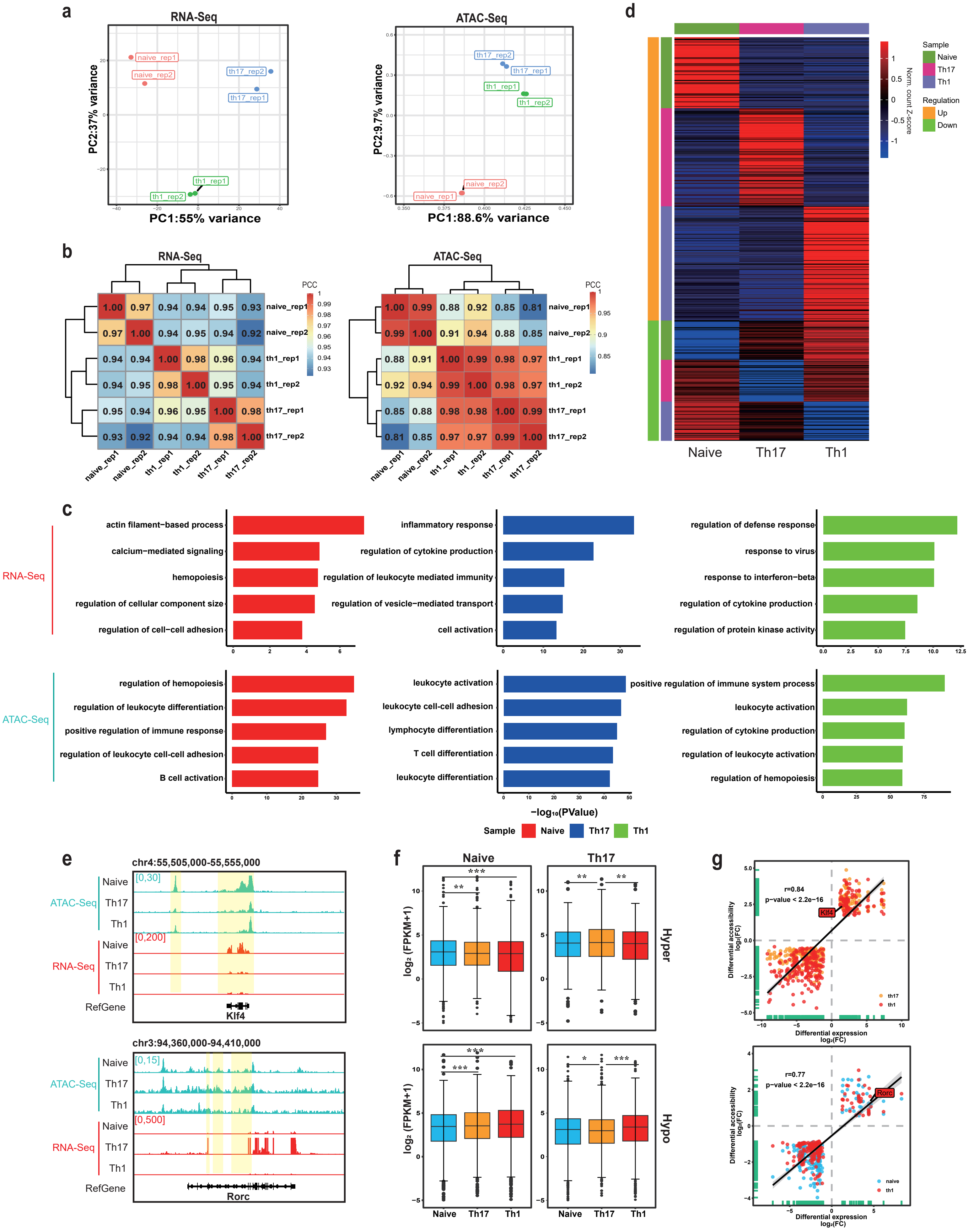

a
RNA-Seq
PC1:55% variance
PC2:37% variance
naive_rep1
naive_rep2
th17_rep2
th17_rep1
th1_rep1
th1_rep2
ATAC-Seq
PC1:88.6% variance
PC2:9.7% variance
b
RNA-Seq
ATAC-Seq
PCC
c
RNA-Seq
ATAC-Seq
actin filament−based process
calcium−mediated signaling
hemopoiesis
regulation of cellular component size
regulation of cell−cell adhesion
inflammatory response
regulation of cytokine production
regulation of leukocyte mediated immunity
regulation of vesicle−mediated transport
cell activation
regulation of defense response
response to virus
response to interferon−beta
regulation of cytokine production
regulation of protein kinase activity
regulation of hemopoiesis
regulation of leukocyte differentiation
positive regulation of immune response
regulation of leukocyte cell-cell adhesion
B cell activation
leukocyte activation
leukocyte cell-cell adhesion
lymphocyte differentiation
T cell differentiation
leukocyte differentiation
positive regulation of immune system process
leukocyte activation
regulation of cytokine production
regulation of leukocyte activation
regulation of hemopoiesis
−log₁₀(PValue)
Sample Naive Th17 Th1
d
Sample
Naive
Th17
Th1
Regulation
Up
Down
Norm. count Z-score
Naive Th17 Th1
e
chr4:55,505,000-55,555,000
ATAC-Seq
RNA-Seq
Naive
Th17
Th1
[0,30]
[0,200]
RefGene
Klf4
chr3:94,360,000-94,410,000
[0,15]
[0,500]
Rorc
f
Naive
Th17
Hyper
Hypo
log₂ (FPKM+1)
Naive Th17 Th1
g
r=0.84
p-value < 2.2e−16
Klf4
th17
th1
Differential accessibility log₂(FC)
Differential expression log₂(FC)
r=0.77
p-value < 2.2e−16
Rorc
naive
th1

Supplementary Fig. 3 T cell differentiation extensively influences gene expression and chromatin accessibility. a. Scatter plot of RNA-Seq and ATAC-Seq data based on principal component analysis (PCA). Left: PCA of RNA-seq samples is performed using normalized read counts. Right: PCA using read densities at open chromatin peaks across all ATAC-Seq replicates. **b.** Pearson correlation coefficient (PCC) and hierarchical clustering corresponding to RNA-Seq and ATAC-Seq data. **c.** Bar plots show GO terms of cell-type specific up-regulated DEGs of Naive, Th17 and Th1, respectively, and GREAT gene ontology enrichment analysis on biological processes for hyper-accessible regions nearest genes of Naive, Th17 and Th1, respectively. Shown are the top five enriched ontologies. **d.** Heatmap of cell-type specific differential accessible regions (DARs). **e.** The genomic tracks show ATAC-Seq (green) and RNA-Seq (red) signals around *Klf4* and *Rorc* locus. The cell-type specific hyper-accessible regions are shaded with yellow. **f.** Boxplot of expression of Naive- and Th17-specific hyper- or hypo- accessible regions nearest genes, values are based on $\log_2$(FPKM+1). The boxplots are shown as median (line), interquartile range (box), and minimum to maximum data range (whisker). The p-values by paired Wilcoxon test, * < 0.05, ** < 0.01, *** < 0.001. **g.** Correlation analysis of DARs and their nearest DEGs that were simultaneously up- or down-regulated in Naive and Th17. Each dot represents a gene that is significantly differentially expressed and associated with chromatin accessibilities changed. The Pearson's correlation coefficient (r) and the corresponding P value are shown.

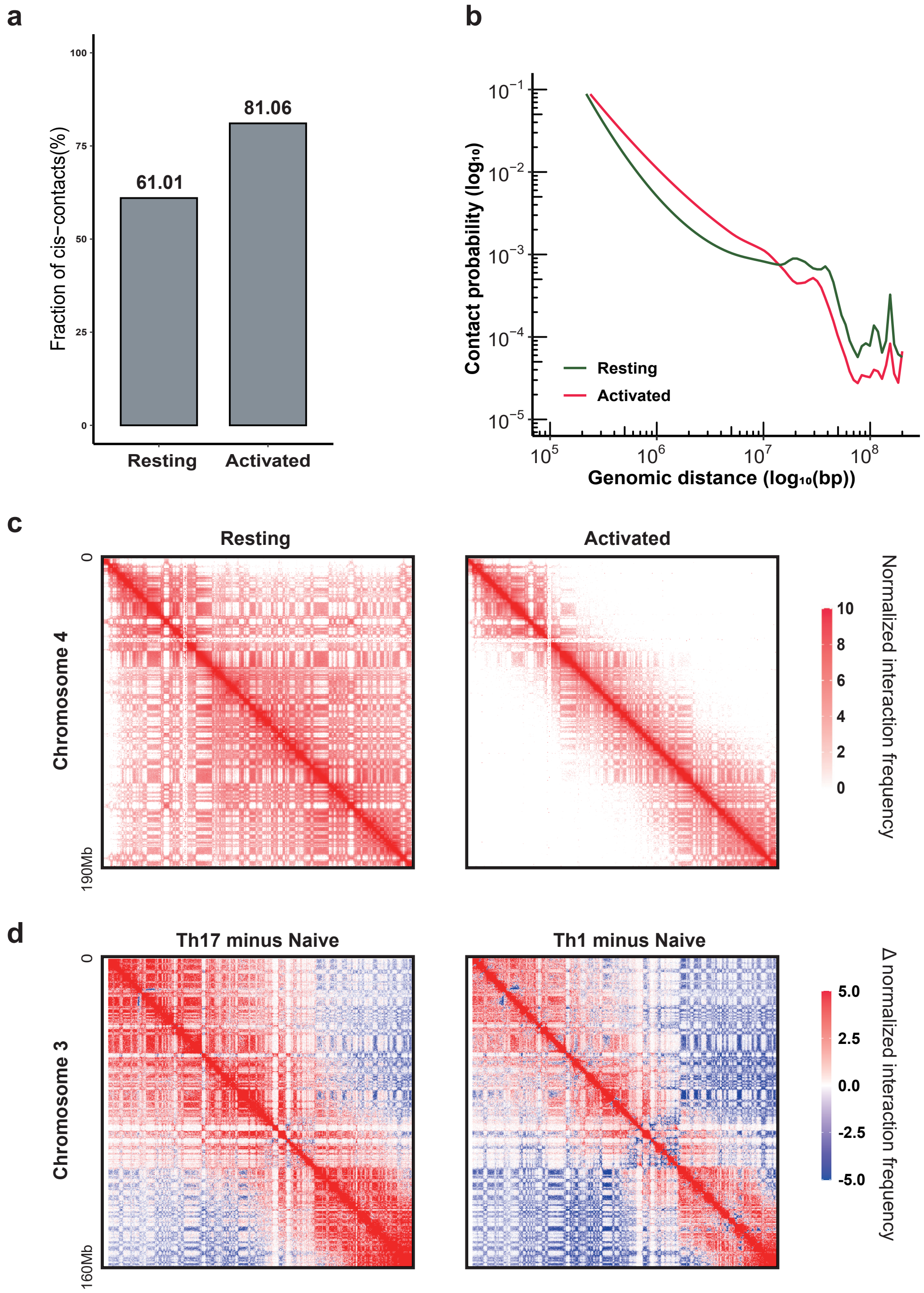

Supplementary Fig. 4 Similar trends in *cis* chromatin contact change in mouse and human T cell activation. a. Fraction of *cis* chromatin contacts per human sample. **b.** The relative contact probabilities (RCP) for Resting and Activated human T cells. **c.** The normalized Hi-C interaction frequencies (100-kb bin, chromosome 4) in Resting and Activated human T cells. **d.** Heatmap shows the differential normalized interaction frequencies at the whole locus between Naive and T helper cells of chromosome 3 in mouse.

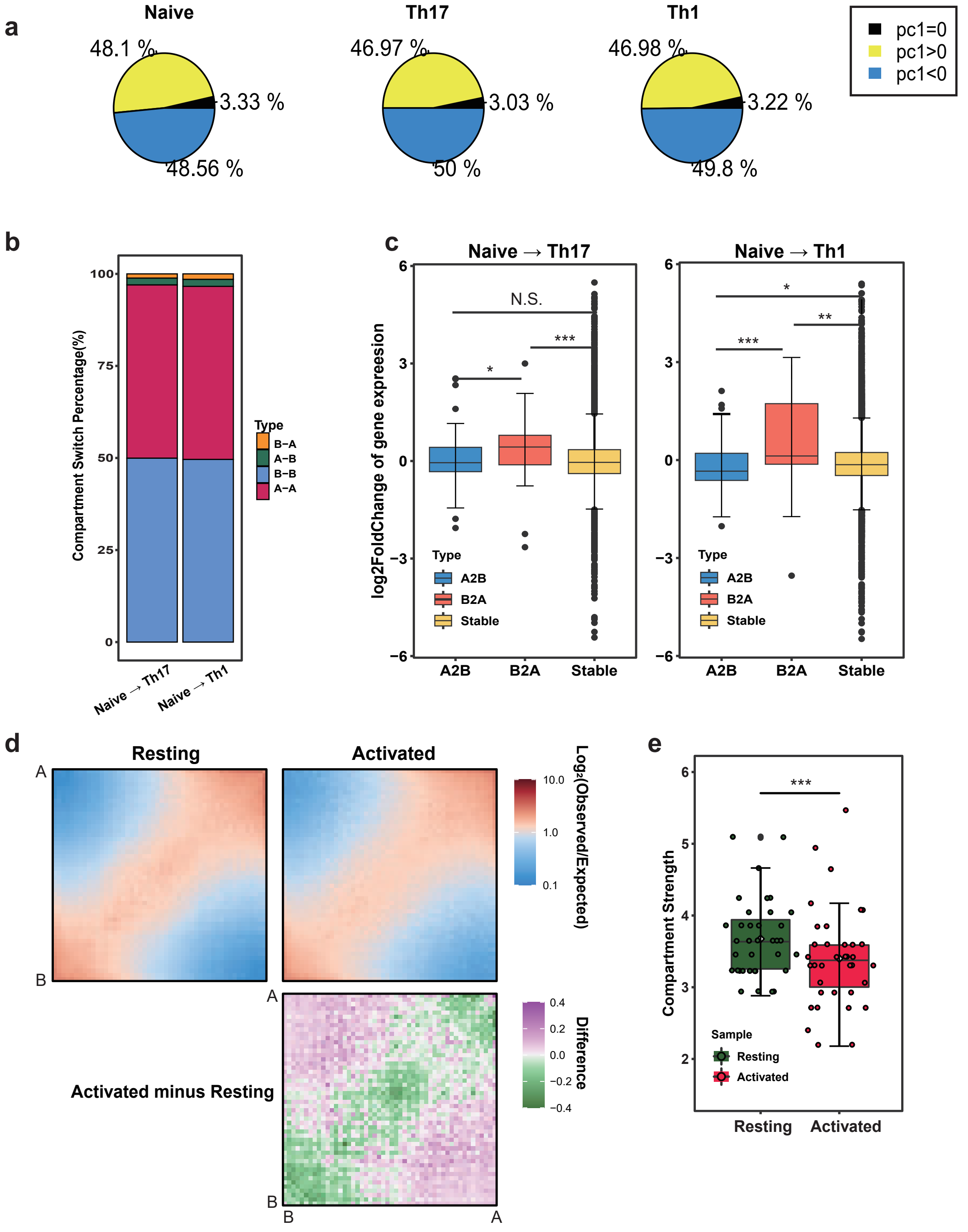

Supplementary Fig. 5 Analysis at compartment level. a. Pie plot of PC1 values distribution. **b.** Fraction of compartment switched regions including A-A, B-B, A-B, B-A. **c.** Boxplots show the distribution of gene expression (FPKM > 3 at least one sample) at regions that exhibit A/B compartment switched upon mouse T cell differentiation. The p-values by unpaired Wilcoxon, N.S. means not significant, * < 0.05, ** < 0.01, *** < 0.001. **d** Saddle-plots and differential saddle plot of the Resting and the Activated are showed. **e.** Boxplot of the human T cells compartmentalization strength per chromosome (dots). The p-values by paired *t*-test, * < 0.05, ** < 0.01, *** < 0.001. The boxplots are shown as median (line), interquartile range (box), and minimum to maximum data range (whisker).

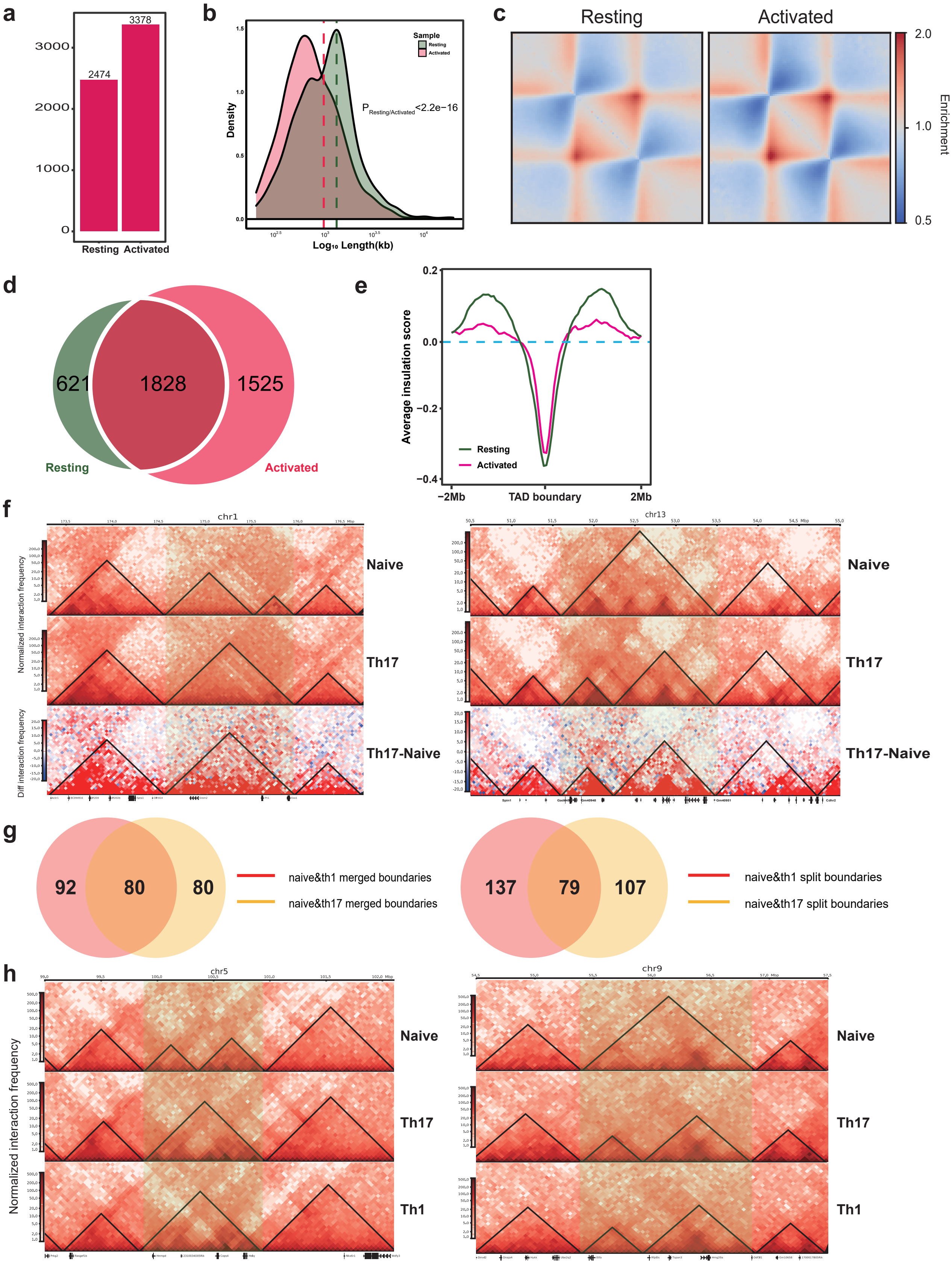

Supplementary Fig. 6 TADs are changed by T cell differentiation. a. TAD number of human T cells (pooled data from two biological replicates of each sample). **b.** Density plot shows human TAD length distribution. The dotted line represents the mean TAD length of Resting and Activated, p-values are calculated by unpaired Wilcoxon test. **c.** Aggregate TAD analysis of human TADs at 40-kb resolution. **d.** The average insulation scores of human T cells at TAD boundaries (± 2 Mb) are shown. **e.** Overlap of human T cells TAD boundaries. **f.** Heatmap of the Merged and Split events between Naive and Th17. The yellow box indicates the area where the event occurs. **g.** Venn diagram showing the number of boundaries of common Merged and Split events that occur in Th17 and Th1. **h.** Regions of common Merged and Split events are displayed. The yellow box indicates the area where the event occurs.

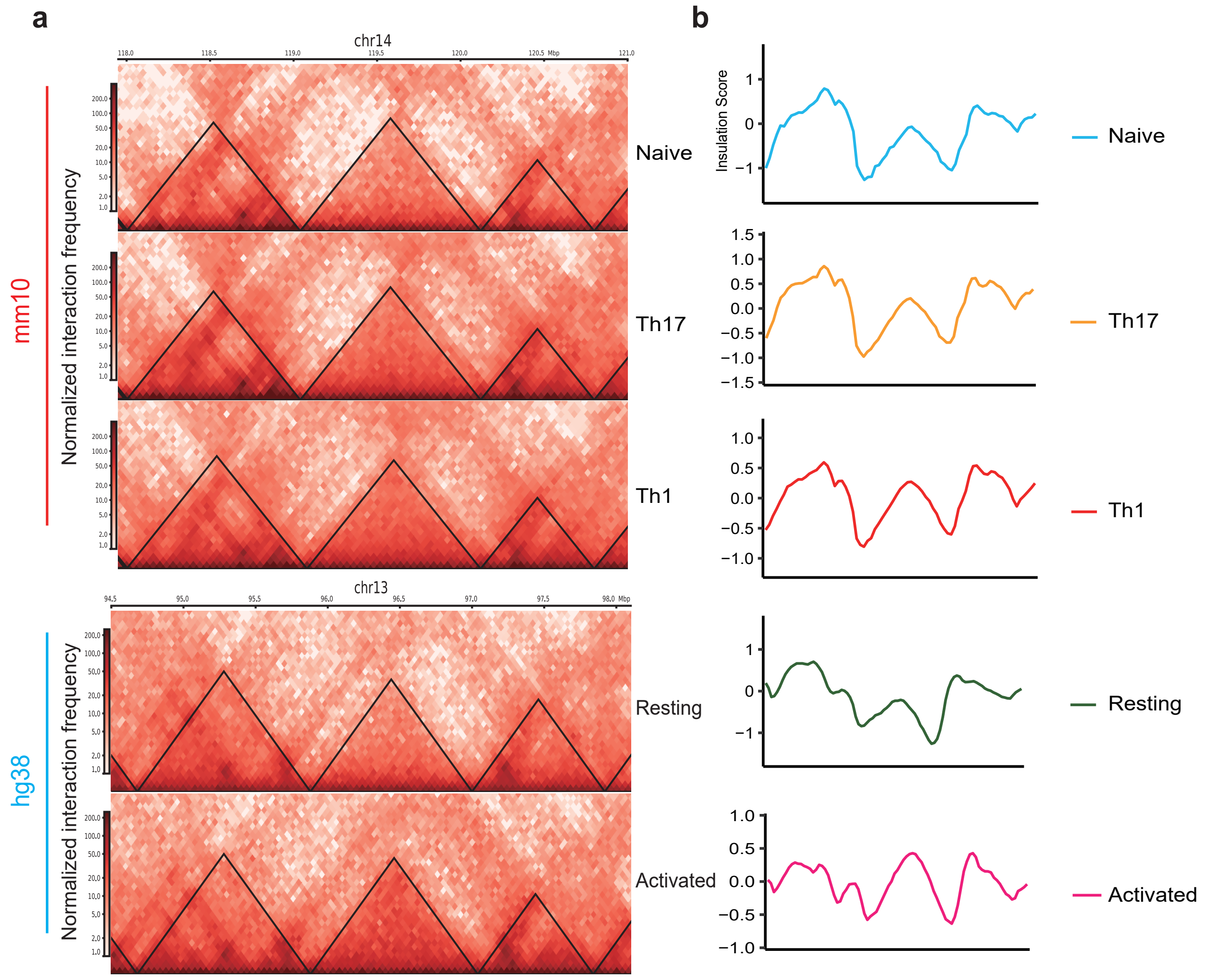

Supplementary Fig. 7 TADs are conserved across T helper cells and species. a. Genome browser shots show TADs over a syntenic region in the mouse and human T cells. Note: the region in humans has been inverted from its normal UCSC coordinates for proper display purposes. **b.** Insulation score line charts corresponding to **a.**

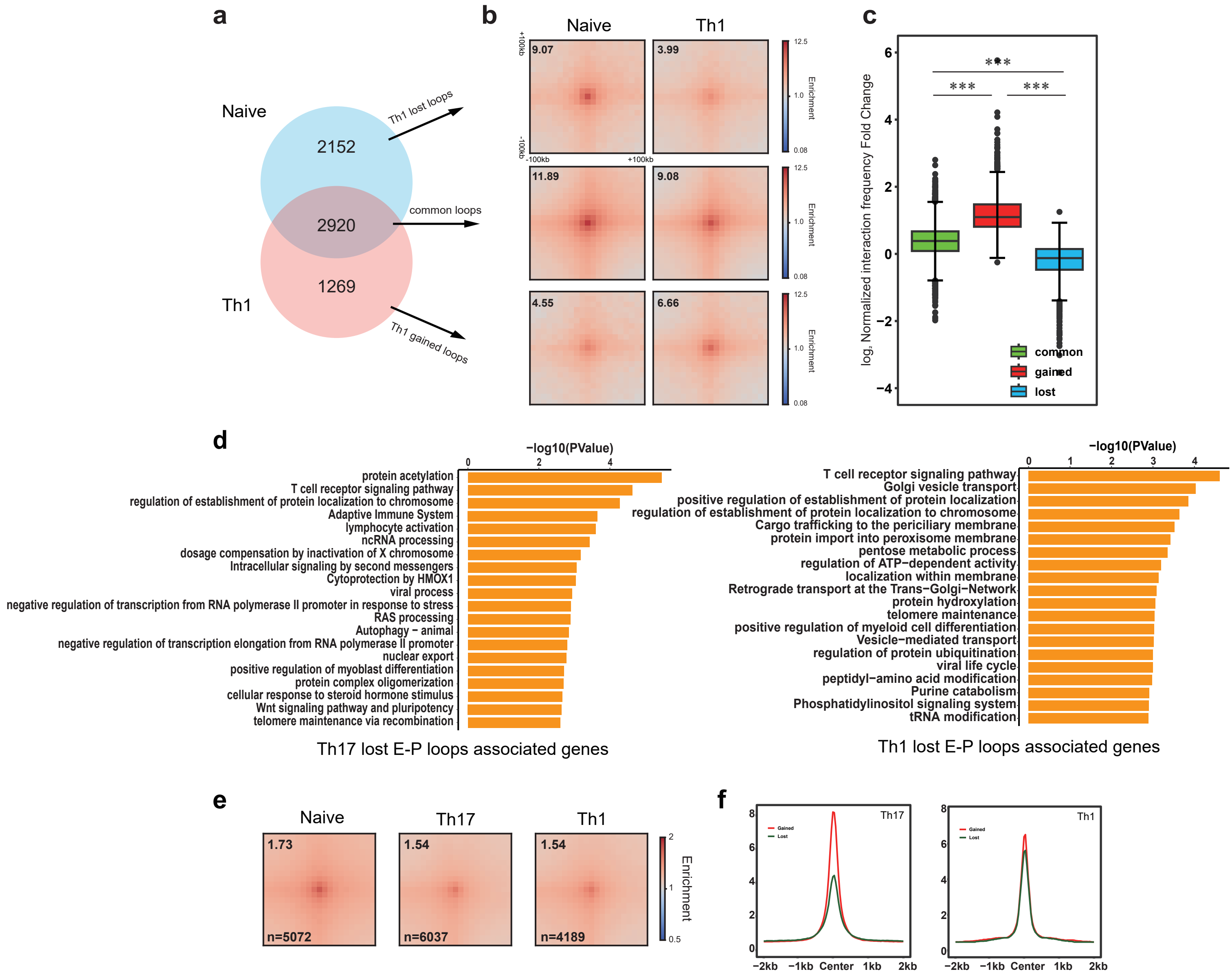

Supplementary Fig. 8 T cell differentiation brings about genome-wide changes in chromatin interactions. a. Venn diagram shows number of loops in Naive and Th1 cells. **b.** Aggregate peak analysis (APA) at 10-kb resolution plots display the average Hi-C signal at chromatin loops that are lost, gained, and common during differentiation from Naive to Th1. The enrichment value in the central pixel is displayed on top left for reference. **c.** The boxplot shows the $\log_2$ normalized interaction frequency fold change (Th1/Naive) for the three groups in **a**. The boxplots are shown as median (line), interquartile range (box), and minimum to maximum data range (whisker). The p-values by unpaired Wilcoxon test, ***< 0.001. **d.** GO enrichment analysis of genes associated with enhancer-promoter lost loops. **e.** Pileups at loops associated with CTCF peaks. Value of the center pixel is shown in the top left corner of each heatmap. The bottom left corner is the number of loops. **f.** Profiles of CTCF density associated with loops of gained or lost in Th1 and Th17 are showed.

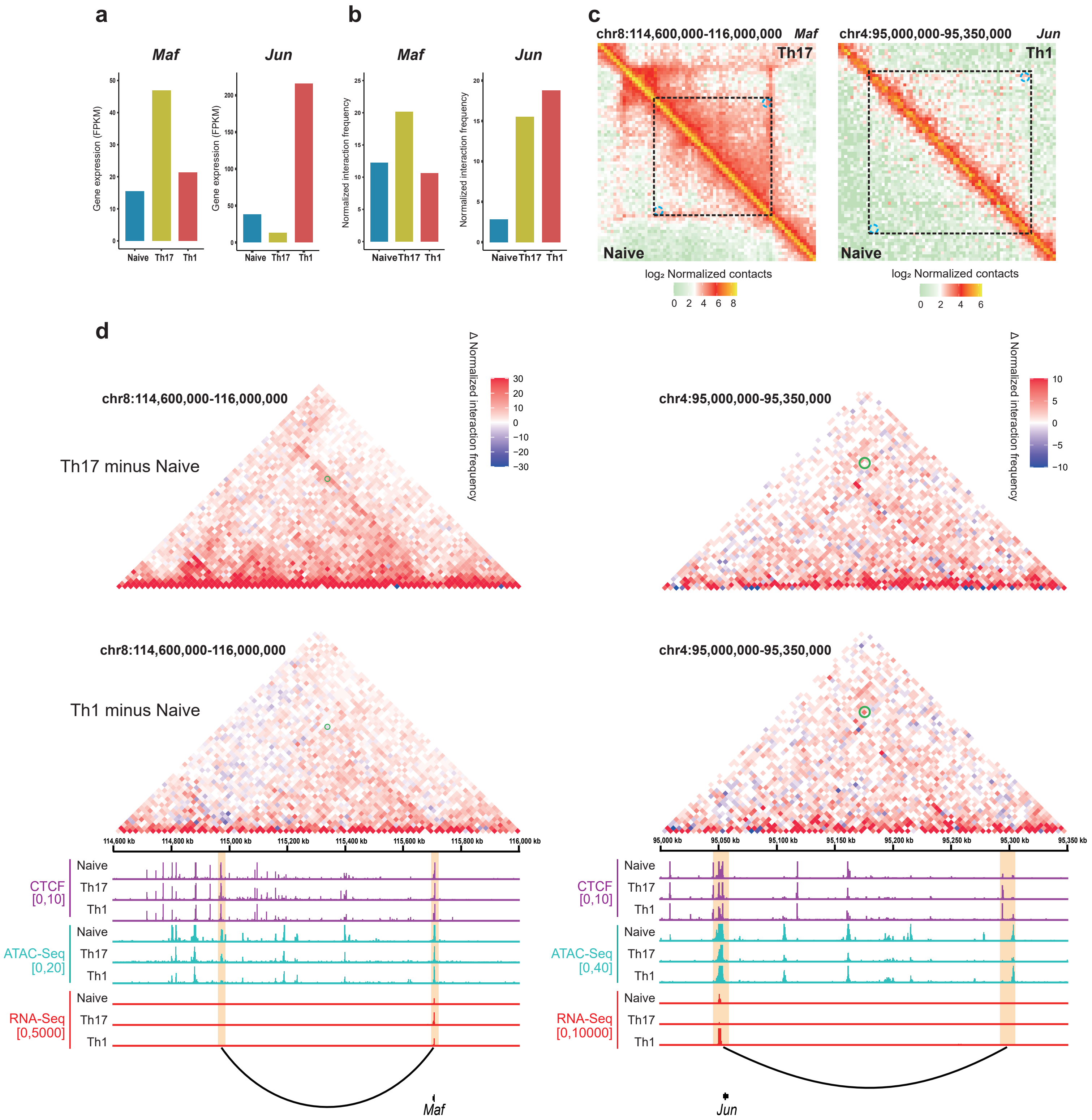

Supplementary Fig. 9 Epigenetic landscapes at *Maf* and *Jun* locus.

a. The gene expression (FPKM) of *Maf* and *Jun*, respectively. **b.** The normalized interaction frequencies of *Maf* and *Jun*, respectively. **c.** The non-subtraction Hi-C maps of *Maf* and *Jun* locus, respectively. The dashed boxes indicate the loop range. The blue dashed circles indicate the Th-specific E-P interaction. **d.** The heatmap shows the differential normalized interaction frequencies at the *Maf* /*Jun* locus between Th17 and Naive , between Th1 and Naive. The green circles indicate the E-P interaction in Th17/Th1 but not in Naive. The bottom tracks show CTCF signals, ATAC-Seq signals, gene expression and Refseq gene. Regions of E-P loop anchors are shown in yellow boxes.

Supplementary Tables

Supplementary Table 1 RNA-Seq and ATAC-Seq data mapping information of T cells

Sample name	Raw reads	Mapped reads	Overall alignment rate	Data type
naive_rep1	31,390,302	28,788,365	91.71%	ATAC-Seq
naive_rep2	20,925,481	20,184,920	96.46%	ATAC-Seq
th17_rep1	24,324,323	22,963,494	94.41%	ATAC-Seq
th17_rep2	25,843,232	24,397,498	94.41%	ATAC-Seq
th1_rep1	27,162,860	25,336,519	93.28%	ATAC-Seq
th1_rep2	25,472,062	23,787,522	93.39%	ATAC-Seq
naive_rep1	85,653,271	79,477,670	92.79%	RNA-Seq
naive_rep2	25,808,588	24,027,795	93.10%	RNA-Seq
th17_rep1	80,170,798	75,320,465	93.95%	RNA-Seq
th17_rep2	23,633,740	21,969,925	92.96%	RNA-Seq
th1_rep1	92,306,818	86,500,719	93.71%	RNA-Seq
th1_rep2	23,876,249	22,159,547	92.81%	RNA-Seq

Supplementary Table 2 Hi-C data mapping information of T cells

Sample name	Total reads	R1 mapped reads	R2 mapped reads	Valid pairs	Valid pairs rmdup	Cis pairs	Trans pairs
naive_rep1	222,551,372	208,307,031	201,992,895	126,607,918	105,051,557	84,373,158	20,678,399
naive_rep2	227,888,752	212,250,305	204,050,689	101,083,694	84,737,669	65,288,591	19,449,078
th1_rep1	268,123,584	230,145,740	223,133,187	128,555,051	106,280,976	81,901,445	24,379,531
th1_rep2	274,450,108	261,319,688	248,344,073	142,059,994	116,447,925	97,417,115	19,030,810
th17_rep1	341,031,863	310,902,120	296,405,225	186,736,158	130,651,561	106,161,301	24,490,260
th17_rep2	244,168,673	231,037,469	219,522,619	141,183,727	118,069,522	96,025,153	22,044,369

Supplementary Codes

```
##### !/usr/bin/env bash
##### sh RNA-Seq.hisat2_htseq-count_stringtie.pipeline.sh fq1 fq2 out_dir out_label .gtf
##### check_strandedness

fq1=$1
fq2=$2
out_dir=$3
out_label=$4
hisat2_index=$5
cpu=$6
gtf=$7

if [ ! -d ${out_dir} ]
then
	mkdir ${out_dir}
else
	echo ${out_dir}" exist!!!"
fi

out_dir=`cd $out_dir|pwd`/${out_dir}
fastp_dir=${out_dir}/1.fastp
hisat2_dir=${out_dir}/2.hisat2
bw_dir=${out_dir}/3.bw
htseq_count_dir=${out_dir}/41.htseq_count
featureCounts_dir=${out_dir}/42.featureCounts
stringtie_dir=${out_dir}/5.stringtie

mkdir -p ${fastp_dir}/0.logs ${fastp_dir}/1.reports ${hisat2_dir}/logs ${bw_dir}
${htseq_count_dir}/logs ${featureCounts_dir}/logs ${stringtie_dir}/logs

#### fastp cut adapter,fastqc
echo `date +%F": "%X`": Start RNA-Seq for ${out_label}!"
echo `date +%F": "%X`": fastp to clean data for ${out_label}!"
cd ${fastp_dir}
fastp -i $fq1 -I $fq2 -o ${out_label}_R1.fastq.gz -O ${out_label}_R2.fastq.gz -j
1.reports/${out_label}.json -h 1.reports/${out_label}.html -w $cpu &>
0.logs/${out_label}.logs

### hisat2 mapping
echo `date +%F": "%X`": HiSAT2 mapping for ${out_label}!"
cd ${hisat2_dir}
hisat2 --dta --no-discordant --no-mixed -p $cpu -x ${hisat2_index} -1
```

```
${fastp_dir}/${out_label}_R1.fastq.gz -2 ${fastp_dir}/${out_label}_R2.fastq.gz -S
${out_label}.sam 2> logs/${out_label}.hisat2.mapping.log

echo `date +%F": "%X`": sam to sorted.bam for ${out_label}!"
sh sam_to_sorted_bam_RNA-Seq.sh ${out_label}.sam $cpu
rm ${out_label}.sam

echo `date +%F": "%X`": sorted.bam to bw for ${out_label}!"
cd ${bw_dir}
bamCoverage -p $cpu -b ${hisat2_dir}/${out_label}.bam -o ${bw_dir}/${out_label}.bw --
normalizeUsing RPKM &> ${out_label}.log

######## count
### htseq-count
echo `date +%F": "%X`": Calculate read counts using htseq-count for ${out_label}!"
cd ${htseq_count_dir}
htseq-count -f bam -r pos -n $cpu -s reverse ${hisat2_dir}/${out_label}.bam ${gtf} >
${out_label}.count 2> logs/${out_label}.log

### featureCounts
echo `date +%F": "%X`": Calculate read counts using featureCounts for ${out_label}!"
cd ${featureCounts_dir}
featureCounts -T $cpu -s 2 -p -B -C -g gene_id -t exon -g gene_id -a $gtf -o
${out_label}.count ${hisat2_dir}/${out_label}.bam &> logs/${out_label}.log

### Stringtie,FPKM
echo `date +%F": "%X`": Calculate FPKM/TPM using Stringtie for ${out_label}!"
cd ${stringtie_dir}
stringtie ${hisat2_dir}/${out_label}.bam -p $cpu --rf -e -G ${gtf} -o ${out_label}.gtf -A
${out_label}_gene_abund.tab 2> logs/${out_label}.stringtie.log

echo `date +%F": "%X`": Finished ${out_label} RNA-Seq!"

################### sam to bam
sam=$1
cpu=$2
flag=${sam%.sam}
bam=${flag}.bam
#sorted_bam=${flag}.sorted.bam

samtools view -bS -@ $cpu $sam|samtools sort -T $flag -@ $cpu -O bam -o - > $bam
samtools index $bam
samtools flagstat $bam > 0.${flag}.txt
```
