# Supplementary material for: Multi-omic analysis reveals dynamic changes of three-dimensional chromatin architecture during T cell differentiation
Source: Commun Biol. 2023 Jul 24;6:773. doi: 10.1038/s42003-023-05141-1 (PMC10366224; doi:10.1038/s42003-023-05141-1)
Supplement: Supplementary file 4 — Reporting Summary [file 42003_2023_5141_MOESM4_ESM.pdf]

nature portfolio

Corresponding author(s): Gang Wei,Ying Li

Last updated by author(s): Jul 4, 2023

Reporting Summary

Nature Portfolio wishes to improve the reproducibility of the work that we publish. This form provides structure for consistency and transparency in reporting. For further information on Nature Portfolio policies, see our Editorial Policies and the Editorial Policy Checklist.

Statistics

For all statistical analyses, confirm that the following items are present in the figure legend, table legend, main text, or Methods section.

n/a	Confirmed	
☐	☒	The exact sample size (*n*) for each experimental group/condition, given as a discrete number and unit of measurement
☐	☒	A statement on whether measurements were taken from distinct samples or whether the same sample was measured repeatedly
☐	☒	The statistical test(s) used AND whether they are one- or two-sided<br>*Only common tests should be described solely by name; describe more complex techniques in the Methods section.*
☐	☒	A description of all covariates tested
☐	☒	A description of any assumptions or corrections, such as tests of normality and adjustment for multiple comparisons
☐	☒	A full description of the statistical parameters including central tendency (e.g. means) or other basic estimates (e.g. regression coefficient) AND variation (e.g. standard deviation) or associated estimates of uncertainty (e.g. confidence intervals)
☐	☒	For null hypothesis testing, the test statistic (e.g. *F*, *t*, *r*) with confidence intervals, effect sizes, degrees of freedom and *P* value noted<br>*Give P values as exact values whenever suitable.*
☒	☐	For Bayesian analysis, information on the choice of priors and Markov chain Monte Carlo settings
☒	☐	For hierarchical and complex designs, identification of the appropriate level for tests and full reporting of outcomes
☐	☒	Estimates of effect sizes (e.g. Cohen's *d*, Pearson's *r*), indicating how they were calculated

Our web collection on statistics for biologists contains articles on many of the points above.

Software and code

Policy information about availability of computer code

Data collection	ALL NCBI GEO datasets listed in Data availability were downloaded using sra toolkit version 3.0.0.
Data analysis	hisat2 version 2.2.1,HTSeq version 0.13.5,stringtie version 2.1.7,R version 4.1.1,deeptools version 3.5.1,bowtie2 version 2.3.5.1.samtoolsversion 1.3.1,bedtools version 2.30.0,MACS2 version 2.2.71,HiC-Pro version 2.11.4,TAD boundary caling by insulation score,HiCExplorerversion 3.7.2,coolpup.py 0.8.7,UCSC liftover,loop calling by HiCCUPS,IGV version 2.12.2.

For manuscripts utilizing custom algorithms or software that are central to the research but not yet described in published literature, software must be made available to editors and reviewers. We strongly encourage code deposition in a community repository (e.g. GitHub). See the Nature Portfolio guidelines for submitting code & software for further information.

Data

Policy information about availability of data

All manuscripts must include a data availability statement. This statement should provide the following information, where applicable:

- Accession codes, unique identifiers, or web links for publicly available datasets
- A description of any restrictions on data availability
- For clinical datasets or third party data, please ensure that the statement adheres to our policy

Al sequencing data created within this tudy was uploaded to NCB1 GEO (https://www.ncbi.nlm.nih,gov/geo and was available under the accession GSE210419. CTCF

ChiP-Sea data were downloaded from GEO for the corresponding samples:CTCF Naive CD4+T cells (GSM3498282), CTCF Th17 cells (GSM3498288) and CTCF Th1 cells (GSM1480825). The Hi-C datasets used for human CD4+T cell 3D genome analysis were from GSE126117.

Human research participants

Policy information about studies involving human research participants and Sex and Gender in Research.

Reporting on sex and gender	n/a
Population characteristics	n/a
Recruitment	n/a
Ethics oversight	n/a

Note that full information on the approval of the study protocol must also be provided in the manuscript.

Field-specific reporting

Please select the one below that is the best fit for your research. If you are not sure, read the appropriate sections before making your selection.

☒ Life sciences ☐ Behavioural & social sciences ☐ Ecological, evolutionary & environmental sciences

For a reference copy of the document with all sections, see nature.com/documents/nr-reporting-summary-flat.pdf

Life sciences study design

All studies must disclose on these points even when the disclosure is negative.

Sample size	No prior sample size determination was conducted. Al experiments were conducted in 2 biological replicates. Statistica testing ensured significant findings.
Data exclusions	No replicates were excluded, and all attempts to replicate were successful.
Replication	All experiments were conducted in 2 biological replicates. For all sequencing data-types, successful replication has been confirmed with Principal Component Analysis.
Randomization	Randomization was relevant to the study, because the difference among three T cell lines was assessed.
Blinding	The investigators were not blinded to sample group allocation, because the difference among three T cell lines was assessed. Sample group assignments were further ensured using Principal Component Analysis on all relevant sequencing data.

Reporting for specific materials, systems and methods

We require information from authors about some types of materials, experimental systems and methods used in many studies. Here, indicate whether each material, system or method listed is relevant to your study. If you are not sure if a list item applies to your research, read the appropriate section before selecting a response.

Materials & experimental systems

n/a	Involved in the study	
☒	☐	Antibodies
☒	☐	Eukaryotic cell lines
☒	☐	Palaeontology and archaeology
☐	☒	Animals and other organisms
☒	☐	Clinical data
☒	☐	Dual use research of concern

Methods

n/a	Involved in the study	
☒	☐	ChIP-seq
☐	☒	Flow cytometry
☒	☐	MRI-based neuroimaging

Animals and other research organisms

Policy information about studies involving animals; ARRIVE guidelines recommended for reporting animal research, and Sex and Gender in Research

Laboratory animals	C57BL/6 mice (1-2 months old) were purchased from Slac Laboratory Animals Inc.
Wild animals	This study did not include wild animals.
Reporting on sex	This study did not apply to only one sex.
Field-collected samples	This study did not include field-collected samples
Ethics oversight	All animal experiments were performed in accordance with protocols approved by the Shanghai Institute of Nutrition and Health Institutional Animal Care and Use Committee.

Note that full information on the approval of the study protocol must also be provided in the manuscript.

Flow Cytometry

Plots

Confirm that:

☒ The axis labels state the marker and fluorochrome used (e.g. CD4-FITC).

☒ The axis scales are clearly visible. Include numbers along axes only for bottom left plot of group (a 'group' is an analysis of identical markers).

☒ All plots are contour plots with outliers or pseudocolor plots.

☒ A numerical value for number of cells or percentage (with statistics) is provided.

Methodology

Sample preparation	Mouse naive CD4+ T cells were isolated from pooled lymph nodes and spleens by using Miltenyi Biotec's isolation kit. Th1 and Th17 cells were induced from naive CD4+ T cell by specific antibodies and cytokines.
Instrument	Samples were analyzed on BD Accuri C6 Flow cytometer or BD FACS Celesta flow cytometer.
Software	Data from Accuri C6 were collected and analyzed by CFlow Plus software.Data from FACS Celesta were analyzed by using FlowJo software.
Cell population abundance	Purity of naive CD4+ T cell were about 88%-90%. IL-17A positive population was about 40% in Th17 cells. IFN-γ positive population was about 60% in Th1 cells.
Gating strategy	The collection of FACS data was conducted by the technician of Cell analyze Center of CEMC,CAS. Naive CD4 Cell purity was assessed by staining for surface expression of CD4, CD44 and CD62L markers. For differentiated Th17 cells, the expression of IL-17A and IFN-γ were measured by intracellular staining. FOXP3 expression was also measured according to the manufacture's protocol of FOXP3 staining kit. For differentiated Th1 cells, the expression of IFN-γ and IL-4 were measured by intracellular staining.

☒ Tick this box to confirm that a figure exemplifying the gating strategy is provided in the Supplementary Information.
